# A pan-cancer landscape of IGF2BPs and their association with prognosis, stemness and tumor immune microenvironment

**DOI:** 10.3389/fonc.2022.1049183

**Published:** 2023-01-04

**Authors:** Wei Shao, Hui Zhao, Shoudu Zhang, Qian Ding, Yugang Guo, Kaiqi Hou, Yunchao Kan, Fan Deng, Qian Xu

**Affiliations:** ^1^Henan Provincial Engineering Laboratory of Insects Bio-reactor, Nanyang Normal University, Nanyang, Henan, China; ^2^The Department of Science and Technology, Zhengzhou Revogene Ltd, Zhengzhou, Henan, China; ^3^Department of Cell Biology, School of Basic Medical Sciences, Southern Medical University, Guangzhou, Guangdong, China

**Keywords:** insulin-like growth factor 2(IGF2) messenger RNA, pan-cancer, prognosis signature, glioma stem cells, tumor microenvironment

## Abstract

**Background:**

The human insulin-like growth factor 2 mRNA binding proteins 1–3 (IGF2BP1–3, also called IMP1–3) play essential roles in mRNA regulation, including its splicing, translocation, stability, and translation. However, knowledge regarding the involvement of IGF2BPs in tumor immunity and stemness across cancer types is still lacking.

**Methods:**

In this study, we comprehensively analyzed pan-cancer multi-omic data to determine the correlation of IGF2BPs mRNA and protein expression with various cancer parameters such as mutation frequency, prognostic value, the tumor microenvironment (TME), checkpoint blockade, tumor immune infiltration, stemness and drug sensitivity. Validation of the expression of IGF2BPs in cancer samples and glioma cells were performed by quantitative real-time (qRT)-PCR, and immunofluorescence staining. Investigation of the functional role of IGF2BP3 in glioma stem cells(GSCs) were performed by sphere formation, cytotoxicity, transwell, and wound healing assays.

**Results:**

We found that IGF2BP1 and 3 are either absent or expressed at very low levels in most normal tissues. However, IGF2BP1-3 can be re-expressed in a broad range of cancer types and diverse cancer cell lines, where their expression often correlates with poor prognosis. Immunofluorescence staining and qRT-PCR analyses also showed that the expression of IGF2BP2 and IGF2BP3 were higher in cancer tissues than that in adjacent normal tissues. Moreover, IGF2BPs are associated with TME and stemness in human pan-cancer. Remarkably, IGF2BP3 participated in the maintenance and self-renewal of glioma stem cell (GSCs). Knockdown of IGF2BP3 attenuated GSC and glioma cell proliferation, invasion, and migration.

**Conclusions:**

Our systematic pan-cancer study confirmed the identification of IGF2BPs as therapeutic targets and highlighted the need to study their association with stemness, and the TME, which contribute to the cancer drug-discovery research. Especially, preliminary studies demonstrate the IGF2BP3 as a potential negative regulator of glioma tumorigenesis by modulating stemness.

## Introduction

The insulin-like growth factor-2 messenger (m)RNA-binding proteins 1–3 (IGF2BP1–3) belong to a family of highly conserved single-stranded-RNA-binding proteins, which regulate RNA processing at multiple levels, including its localization, translation, and stability (1). N6-methyladenosine (m6A) is the newest and currently most studied mRNA modification in eukaryotes, because of its implication in the progression of several types of cancer (2). IGF2BPs act as m6A readers by recognizing and regulating the m6A modification of target mRNAs, which is crucial for their oncogenic function (3–5). Several studies have shown that IGF2BPs are associated with the progression of cancers such as hepatocellular carcinoma (HCC) (6), non-small cell lung cancer (NSCLC) (7), colorectal cancer (8), and gastric cancer (9). Dysregulation of IGF2BPs may cause abnormal gene expression and therefore help the malignant phenotypes of cancer cells (10–12). For example, the activation of actin beta (*ACTB*) mRNA translation occurs *via* the Src-directed tyrosine phosphorylation of the linker region connecting K homology (KH)-domains 2 and 3 of IGF2BP1 (13). Moreover, the long non-coding (lnc)RNA *LINRIS* stimulates aerobic glycolysis in colorectal cancer (COAD) by stabilizing IGF2BP2 *via* the inhibition of IGF2BP2 K139 ubiquitination (14). Meanwhile, IGF2BP3 promotes the suppression of ZO-1 signaling by micro (mi)R191-5p, which increases the invasiveness of HCC cells (15).

Tumor progression is controlled by factors associated with tumor cells and those associated with infiltrating immune cells and the TME (16). The TME is created by tumor cells, immune cells, stromal cells, cancer stem cells (CSCs), and extracellular components, which all play important roles in tumor progression and drug resistance (17, 18). The most recent studies suggest that CSCs are an important component of the TME, with multipotent differentiation potential and can regulate tumor occurrence, development, recurrence, metastasis, and drug resistance (19). It has also been suggested that the activities of CSCs and immune cells represent the vital link between the TME and cancer (20, 21). CSC properties are controlled by the cellular and extracellular matrix components of the TME (22). Recent studies have demonstrated that m6A regulators, such as METTL3/14, ALKBH5, FTO, YTHDF2, IGF2BP1/2, and HNRNPA2B1 participate in the modulation of GSCs (23, 24). For example, the inhibition of IGF2BP1 expression, reduces the proliferation of leukemia stem cells (LSCs), while promoting their differentiation and apoptosis. In addition, IGF2BP2 has been reported to participate in the regulation of CSCs in acute myeloid leukemia (AML) (24). Emerging evidence suggests that the m6A regulators (such as METTL3, HNRNPC, YTHDF1, FTO, and IGF2BP3) modulate the response to immunotherapy *via* TME (comprising immune cells, checkpoints, and cytokines) remodeling (25). Although several studies have highlighted the functional importance of IGF2BPs in the crosstalk between the TME and CSCs, their role in tumor immunity and stemness across cancer types, and the underlying mechanisms, remain relatively unknown.

In this study, we performed a comprehensive analysis of multi-omic data from The Cancer Genome Atlas (TCGA), GTEx (Genotype-Tissue Expression), Cancer Cell Line Encyclopedia (CCLE), Gene Expression Omnibus (GEO), Oncomine, and CPTAC (Clinical Proteomic Tumor Analysis Consortium) databases, together with functional experiments to characterize IGF2BPs across multiple cancer types. We focused on the association between IGF2BPs and tumor prognosis, stemness, and the TME, as well as aid in exploring the role the IGF2BP3 in maintenance and self-renewal capacity of GSCs. Collectively, our findings may have important implications for guiding basic research and providing the rationale for developing IGF2BP-targeted anti-cancer therapies.

## Materials and methods

### Data collection and bioinformatics analysis

The UCSC Cancer Genome Browser (https://tcga.xenahubs.net) was used to download the gene expression profiles, MMR gene data, DNA Methylation data, TMB data, MSI data, and clinical attribute information for 33 cancer types. The CCLE database (http://portals.broadinstitute.org/ccle) was used to analyze the mRNA expression profiles of IGF2BPs in 1,100 cell lines. The Sangerbox online platform (http://sangerbox.com/) was used to visualize and analyze IGF2BPs expression differences between pan-cancer tumor tissues and paired normal tissues. We analyzed correlations between the mRNA and protein expression levels of IGF2BPs and clinicopathology using protein expression data from the UALCAN web portal. During the validation process, an analysis of IGF2BPs mRNA transcription levels using data from the Oncomine database (www.oncomine.org) was performed for different types of cancer. GEPIA (http://gepia.cancer-pku.cn/index.html) was used to determine the association between IGF2BPs expression and the prognosis of cancer patients. SangerBox (http://www.sangerbox.com) was used to generate forest plots. The log-rank P-value (Kaplan–Meier method) and hazard ratio (HR) with a 95% confidence interval (95% CI) were calculated. The correlations between genes and six immune cells were analyzed using the Spearman or partial Spearman method. The stemness features from TCGA tumor samples were extracted and used to measure the stem-cell-like features of tumor cells. Estimated immune and stromal scores were calculated using the ESTIMATE algorithm (Estimation of STromal and Immune cells in Malignant Tumor tissues with Expression data). This algorithm was applied to calculate tumor purity and the ESTIMATE, immune, and stromal scores of each sample using the R package “estimate” (26). Six immune subtypes were defined to measure immune infiltrates in TME (27). We used ANOVA models to examine the relationship between SEMA3 expression and immune infiltrate types in tumor microenvironments using data from the TCGA pan cancer database. Meanwhile, an analysis of multiple cancers was conducted using the SangerBox online platform to determine whether IGF2BPs expression was correlated with 10 immune cell types in the TME. CellMiner (http://discover.nci.nih.gov/cellminer/) was used to predict the correlation between IGF2BP1/2 expression and the anti-cancer drug response.

### Sample collection

All tumor and paired normal tissues were collected from patients who underwent surgery at the Nanyang Central Hospital (Nanyang, Henan, P.R. China) from 2021 to 2022; tissue samples (stored in paraffin) were collected from patients with esophageal carcinoma (ESCA), stomach adenocarcinoma (STAD), or COAD, and blood was collected from patients with GBM. The clinical information related to the samples is summarized in **Supplementary Tables 1, 2**. The histological diagnosis was established according to the World Health Organization (WHO) classification criteria. All participants were suitably informed and agreed to take part in the study. This study was approved by the Ethics Committee of the Nanyang Central Hospital. All the patients (or their guardians) participating in this study provided informed consent.

### Immunofluorescence staining

Immunofluorescence staining was performed to detect the expression of IGF2BP2 and IGF2BP3. In brief, the tissue samples were deparaffinization, blocked at room temperature for 60 min with a blocking solution (5% BSA/0.1% Triton X-100 PBS), and stained with the primary anti-IGF2BP2 (1:200; 11601-1-AP; Proteintech) and anti-IGF2BP3 (1:200; 14642-1-AP; Proteintech) antibodies. The samples were then washed with Tris-buffered saline for 30 min, and incubated with the secondary goat anti-rabbit IgG H&L (Alexa Fluor^®^ 568) (1:200, ab175471; Abcam) and goat anti-mouse IgG H&L (Alexa Fluor 488) (1:200, ab150113; Abcam) antibodies for 30 min at 37 °C in the dark. After washing in PBS, DAPI staining was performed for 5 min, to counterstain the nuclei (28). Finally, the samples were sealed in 50% glycerin and imaged on a fluorescence microscope (DP72, OLYMPUS, Japan).

### Total RNA extraction and quantitative real-time (qRT)-PCR

The collected fresh cancer tissues and paired paracancerous tissues were immediately placed in RNA later and stored at −80°C for RNA extraction. The peripheral blood samples were immediately stored in blood RNA storage tubes (BioTeke Corporation, Beijing, China). Total RNA was isolated with TRIzol reagent (Invitrogen) and then cDNA was synthesized using the M5 Superplus RT-qPCR Kit with gDNA (Mei5 Biotechnology, Co., Ltd, Beijing, China). qRT-PCR was conducted on the CFX96TM System (Bio-Rad, USA) using the 2^−ΔΔCT^ method for comparative quantification (29). GAPDH served as the internal reference gene. The following primers were used: IGF2BP2-F: 5′-ACCAGTGCAGAAGTCATCGT-3′, IGF2BP2-R: 5′-GGAAGGGCTACATTCATCCGTT-3′. IGF2BP3-F: 5′-AGGCGCTTTCAGGTAAAATAG-3′. IGF2BP3-R: 5′-TAAACTATCCAGCACCTCCC-3′.

### Cells culture

The human glioma U251 and HS683 cell lines were obtained from the Chinese Academy of Medical Sciences (Shanghai, China). The cell lines were cultured in Dulbecco’s modified Eagles medium (DMEM) containing 10% fetal bovine serum (FBS) and 1% penicillin–streptomycin solution at 37°C, 5% CO_2_, 95% humidity.

### Lentiviral transduction of glioma cells

A lentivirus‐based packaging system was developed using the plvx-shRNA2-ZSGreen-T2A-puro lentiviral overexpression vector and the pSIH1‐H1‐copGFP lentiviral short hairpin RNA (shRNA) fluorescent expression vector. The shRNAs for IGF2BP3 and their shRNA positive controls (sh‐NC) were constructed by Oligobio (Beijing, China). Briefly, after reaching 30%–50% confluency, the U251 and HS683 cells were transduced with lentiviral particles at a multiplicity of infection (MOI) of 50. After 12 h of transduction, over 95% of the cells were still viable. The culture medium was discarded and replaced with fresh complete culture medium. Following transduction, the HS683 and U251 cells were cultured for 24 h at 37°C prior for use in subsequent experiments, as previously described (30).

### Sphere formation assay

The cells were grown until 80% confluence, then digested with trypsin into a suspension of single cells. Thereafter, 10^3^ cells were resuspended in 1 mL of serum-free DMEM-F12 medium containing epidermal growth factor (EGF, 20 ng/mL, basic fibroblast growth factor (b-GFG, 20 ng/mL), and a B27 supplement. The cells were then transferred into a 24-well ultra-low-attachment plate (Corning, USA). After 8–10 days of incubation at 37°C, the spheres were quantified under a light microscope (×100), as previously described (31).

### Cytotoxicity analysis

Cell Counting Kit-8 (CCK-8; Sigma-Aldrich, St. Louis, MO, USA) was used to determine cell viability during cell proliferation, according to the manufacturer’s protocol. After lentiviral transduction, 10 mL of CCK-8 reagent (5 mg/mL) was added to each well, and the cells were incubated for 2 h at 37°C. A microplate spectrophotometer was used to measure absorbance at 450 nm.

### Invasion assay and wound healing assay

The cell invasion assay was conducted in Transwell chambers (Corning, Tewksbury, MA, USA) coated with Matrigel (dilution 1:8; BD Biosciences, Bedford, MA, USA) and polycarbonate membranes. The upper chamber contained 5×10^4^ cells in 120 μL serum-free medium, while the lower chamber contained in 600 μL of medium supplemented with 10% FBS. Lentiviral transduction was then performed. After fixation with formaldehyde, crystal violet was used to stain cells adhering to the lower Transwell chamber. Images were captured using an inverted microscope (AMG, USA).

To perform the wound healing assay, U251 and HS683 cells were plated in the 6-well plates. When the cells had reached 80% confluency, the monolayer of cells was scraped off using a 10 μL sterile pipette tip followed by lentiviral transduction. Thereafter, fluorescence images were obtained with an inverted fluorescence microscope (EVOS, AMG, USA). A quantitative analysis of cell migration was performed using Image J software version 1.46 (National Institutes of Health, Bethesda, MD, USA).

### Statistical analysis

Plots were created using R software version 3.6.1 (https://www.r-project.org/) with packages ggplot2, pheatmap, corrplot, or survminer where appropriate. Statistical analysis was performed using GraphPad Prism version 8.0 (Graphpad Software, CA, USA). All experiments were performed at least three times in triplicate. Statistically significant differences were defined as those with a P-value less than 0.05.One-way analysis of variance (ANOVA) with the Student-Newman-Keul’s *post-hoc* test was used to determine whether there was a statistically significant difference between the two groups.

## Results

### IGF2BP*s* are highly expressed in several types of cancer

**Figure 1:**
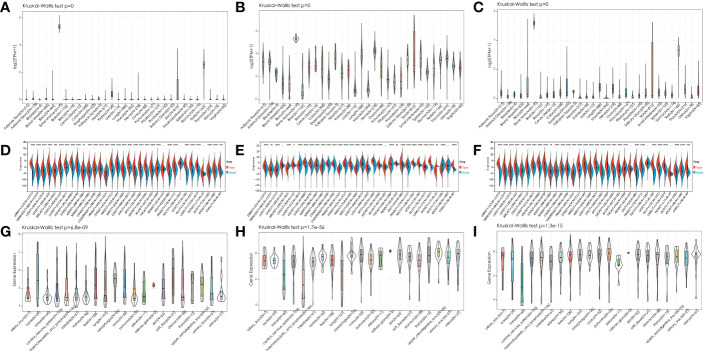
Expression pattern of IGF2BPs in human cancers. **(A-C)** Expression pattern of IGF2BPs in diverse normal tissues (data from GTEx). **(D-F)** Expression pattern of IGF2BPs in GTEx normal, TCGA normal, and TCGA cancer tissues. **(G-I)** Expression pattern of IGF2BPs in diverse cancer cells (data from CCLE). *P < 0.05, **P < 0.01, ***P < 0.001, ****P < 0.0001.

**Figure 2:**
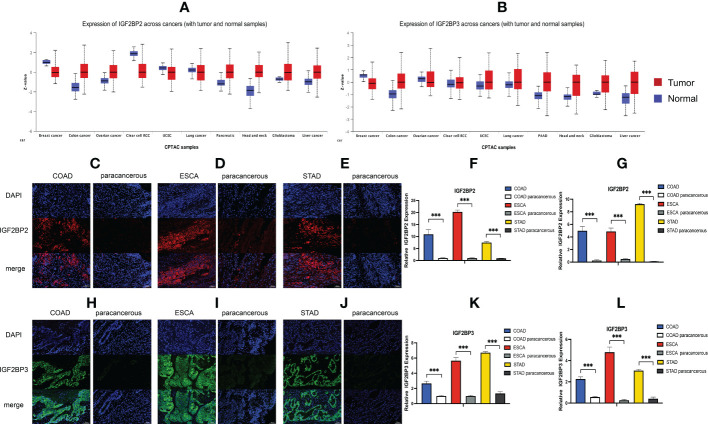
Expression level of IGF2BP2 and IGF2BP3 in COAD, ESCA and STAD cancer tissues. **(A, B)** IGF2BP2/3 protein expression levels among normal tissue and primary tissue were compared based on the CPTAC dataset. Immunofluorescence of IGF2BP2 (red) in validated cancer tissues and adjacent tissues. Representative immunofluorescence images are presented in validated cancer tissues **(C)** COAD, **(D)** ESCA and **(E)** STAD and adjacent tissues. **(F)** Immunoreactivity of IGF2BP2 was quantified. **(G)** The mRNA expression of IGF2BP2 in validated cancer tissues and adjacent tissues by RT-qPCR. Immunofluorescence of IGF2BP3 (green) in validated cancer tissues and adjacent tissues. Representative immunofluorescence images are presented in validated cancer tissues **(H)** COAD, **(I)** ESCA and **(J)** STAD and adjacent tissues. **(K)** Immunoreactivity of IGF2BP3 was quantified. **(L)** The mRNA expression of IGF2BP3 in validated cancer tissues and adjacent tissues by RT-qPCR. Data are mean ± SEM (n = 3 per group). *P < 0.05, **P < 0.01, ***P < 0.001.

We first analyzed the expression of IGF2BPs genes in 31 normal tissues utilizing the GTEx database. As shown in **Figures 1A–C**, IGF2BP1 and 3 are either absent or expressed at very low levels in most normal tissues. Some differences can also be seen that the IGF2BP1 and IGF2BP2 expression were higher in the bone marrow, skin, and testes, compared with that in other tissues. IGF2BP2 was generally more highly expressed than IGF2BP1 or IGF2BP3 in normal tissues. However, by integrating the GTEx and TCGA datasets, IGF2BP1-3 can be re-expressed in a broad range of cancer types and diverse cancer cell lines (**Figures 1D–I**), Specifically, IGF2BP1 expression was significantly higher in most of the tumor tissues (n = 30/34) and significantly lower in prostate cancer (PRAD) (P < 0.05) than that in normal tissues (**Figure 1D**); IGF2BP1 expression was similar between rectal cancer (READ) (P > 0.05), testicular cancer (TGCT) (P > 0.05), and pheochromocytoma & paraganglioma (PCPG) (P > 0.05) tumor tissues and normal tissues. The expression of IGF2BP2 was higher in most of the tumor tissues (n = 28/34) and lower in adrenocortical cancer (ACC) (P < 0.05), PRAD (P < 0.05) and KIPAN (P < 0.05), compared to normal tissues (**Figure 1E**); IGF2BP2 expression in endometrioid cancer (UCEC) (P > 0.05), bladder cancer (BLCA) (P > 0.05), and kidney chromophobe (KICH) (P > 0.05) tumors was similar to that in normal tissues. IGF2BP3 expression was higher in most of the tumor tissues (n = 30/34) and lower in PRAD (P < 0.05) (**Figure 1F**); IGF2BP3 expression in READ (P > 0.05), TGCT (P > 0.05), and PCPG (P > 0.05) tumors was similar to that of normal tissues. We further evaluated IGF2BPs expression in 1,156 cancer cell lines using data from the CCLE database. This analysis revealed that IGF2BPs were generally more highly expressed in cancer cell lines (**Figures 1G–I**). In addition, the Oncomine database was used to verify the high expression of IGF2BP2 and IGF2BP3 in cancer tissues. We observed that IGF2BP2 and IGF2BP3 expression was markedly upregulated in the central nervous system (CNS), lung, gastric tract, head, neck, pancreas, and lymphatic system cancers (**Figure S1A**). Meanwhile, compared with IGF2BP1 and IGF2BP3, IGF2BP2 expression exhibited striking intra- and inter-tumor heterogeneity. For example, some tumors such as bile duct cancer (CHOL), head and neck cancer (HNSC), lung squamous cell carcinoma (LUSC), GBM, STAD, THCA, and BLCA exhibited high IGF2BP2 expression levels, while KIRC, breast cancer (BRCA), and UCEC were characterized by low levels of IGF2BP2 expression (**Figure S1B**). Next, we analyzed the protein levels of IGF2BP2 and IGF2BP3 in different tumor types and normal tissues using the CPTAC dataset. Compared with adjacent normal tissues, IGF2BP2 expression was higher in LUAD, LICH, GBM, HNSC, PAAD, OV, and COAD tissue (**Figure 2A**), while IGF2BP3 expression was upregulated in BRCA, CESC, LUAD, LICH, UCEC, GBM, HNSC, PAAD, OV and COAD tissue (**Figure 2B**), which were be validated in COAD, ESCA, and STAD by immunofluorescence staining and qRT-PCR analysis (**Figures 2C–L**). The result shown that the expression of both protein and RNA levels of IGF2BP2 and IGF2BP3 were up-regulated in COAD, ESCA, and STAD cancer tissues compared with paracancerous.

### Prognostic potential and clinicopathology of IGF2BPs expression in human pan-cancer

**Figure 3:**
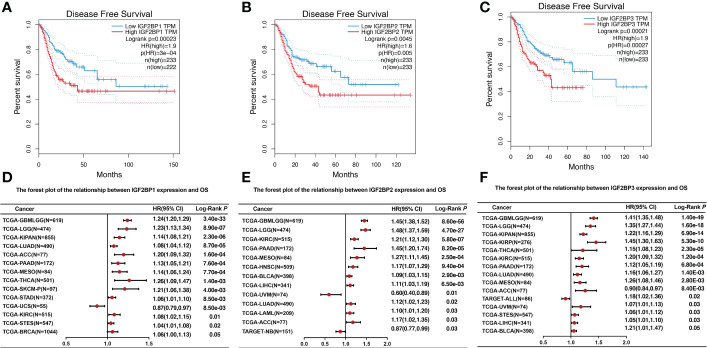
The relationship between IGF2BPs expression level and overall survival. **(A-C)** Kaplan–Meier survival curves comparing the high and low expression of IGF2BPs in 33 cancer types. **(D)** The forest plot of the relationship between IGF2BP1 expression and OS across 13 tumors. **(E)** The forest plot of the relationship between IGF2BP2 expression and OS across 12 tumors. **(F)** The forest plot of the relationship between IGF2BP3 expression and OS across 15 tumors.

To further investigate the prognostic potential of IGF2BPs in cancers, we investigated the link between IGF2BPs expression and pan-cancer prognosis. As shown in **Figures 3A–C**, the difference in overall survival (OS) time between patients with high and low expression levels of IGF2BP1 (HR = 2, P = 0, n = 4701), IGF2BP2 (HR = 2.4, P = 0, n = 4740), and IGF2BP3 (HR = 3.5, P = 0, n = 4680). For instance, high IGF2BP1 expression level was associated with the poor prognosis of patients with ACC (HR = 1.20, P < 0.001), BRCA (HR = 1.06, P <0.05), KIPAN (HR = 1.14, P <0.001), KIRC (HR = 1.08, P <0.01), LUAD (HR = 1.08, P <0.001), LGG (HR = 1.23, P <0.001), PAAD (HR = 1.13, P <0.001), STAD (HR = 1.06, P <0.01), MESO (HR = 1.14, P <0.001), SKCM (HR=1.21, P <0.01), STES (HR = 1.04, P <0.001), and THCA (HR = 1.26, P <0.01) (**Figure 3D**); high expression of IGF2BP2 predicted poor prognosis in patients with ACC (HR=1.17, P <0.03), BLCA (HR=1.09, P <0.01), KIRC (HR = 1.21, P <0.001), LGG (HR = 1.48, P <0.001), HNSC (HR=1.17, P <0.001), LUAD (HR = 1.12, P <0.02), LAML (HR = 1.10, P <0.03), LICH (HR = 1.11, P <0.01), PAAD (HR = 1.45, P <0.001), and MESO (HR = 1.27, P <0.001) (**Figure 3E**); and high expression of IGF2BP3 was a risk factor for poor prognosis in patients with ACC (HR = 0.90, P <0.01), BLCA (HR = 1.21, p<0.05), KIPAN (HR = 1.22, P <0.001), KIRC (HR=1.20, P <0.001), KIRP (HR = 1.45, P <0.001), LGG (HR = 1.35, P <0.001), LUAD (HR=1.16, P <0.01), LICH (HR=1.05, P <0.03), PAAD (HR = 1.12, P <0.001), MESO (HR = 1.26, P <0.01), UVM (HR = 1.07, P <0.03), STES (HR = 1.06, P <0.03), and THCA (HR = 1.15, P <0.001) (**Figure 3F**). By contrast, increased IGF2BP1 expression was associated with prolonged OS in UCS (HR = 0.87, P <0.01) and TAGET-NB (HR = 0.87, P = 0.03); increased IGF2BP2 expression was associated with prolonged OS in UVM (HR = 0.6, P <0.01); and increased IGF2BP3 expression was associated with prolonged OS in TAGET-ALL (HR = 0.6, P <0.01).

In addition, we examined the relationship between IGF2BPs expression levels and patient clinicopathological characteristics (cancer stage, age, ethnicity, and sex) using a TCGA dataset. As shown in **Figure S2**, there was significant difference in the expression of IGF2BPs between four age ranges (**Figures S2A–C**, P <0.001), males and females (**Figures S2D–F**, p<0.001), the four cancer stage (**Figures S2G–I**, P <0.001). Of note, no differences in IGF2BPs expression were evident across race (**Figures S2J–L**, P >0.05). These data indicate that high IGF2BPs expression was strongly associated with poor patient outcomes in multiple cancer types.

### Association between IGF2BPs expression and mismatch repair (MMR) gene expression, DNA methylation level, tumor mutational burden (TMB), and microsatellite instability (MSI)

**Figure 4:**
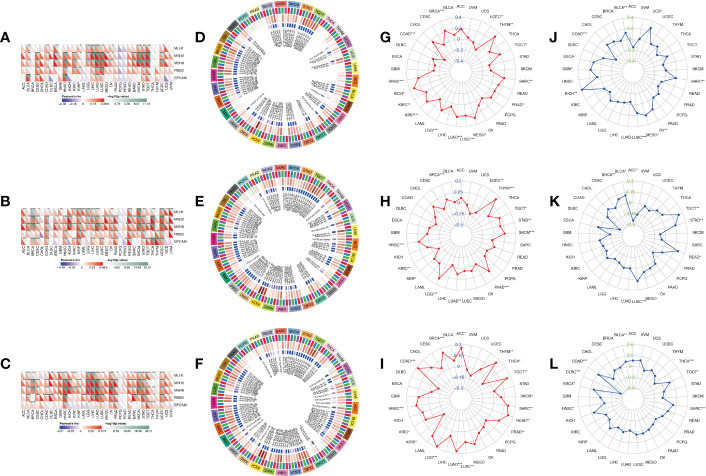
Correlation analysis between IGF2BPs expression and MMR, DNA methylation level, TMB, and MSI in pan-cancer. **(A-C)** The Spearman correlation analysis of IGF2BPs expression with expression levels of five MMR genes across cancers. **(D-F)** The Spearman correlation analysis of IGF2BPs expression with the expression of 4 methyltransferases, Red represents DNMT1, blue represents DNMT2, green represents DNMT3A, and purple represents DNMT3B. **(G-I)** The correlation analysis between IGF2BPs expression and TMB in pan-cancer. **(J-L)** The correlation analysis between IGF2BPs expression and MSI in pan-cancer. *P < 0.05, **P < 0.01, ***P < 0.001.

After determining the prognostic value of IGF2BPs, the association between IGF2BPs and MMR, DNA methylation level, MSI, and TMB in 33 cancers was assessed to determine the potential role of IGF2BPs in tumor progression. First, we evaluated the association between IGF2BPs and the mutation levels of five MMR genes (*MLH1, MSH2, MSH6, PMS2*, and *EPCAM*). The results shown in **Figures 4A–C** revealed that IGF2BP2 and IGF2BP3 expression was highly related to MMR genes expression in multiple cancers, including CESC, COAD, HNSC, KIRC, LGG, LICH, LUAD, LUSC, SKCM, STAD, and TGCT. We next investigated the correlation between the expression of IGF2BPs and that of four DNA methyltransferase genes (*DNMT1, DNMT2, DNMT3A*, and *DNMT3B*). We found that IGF2BPs expression was strongly positively correlated with the expression of DNA methyltransferase genes in multiple cancers, including COAD, KIRC, LGG, LUAD, LUSC, STAD, THCA (but not in PCPG, DLBC, or CHOL) (**Figures 4D–F**, P <0.05), suggesting that IGF2BPs may play a role in tumor progression by mediating DNA repair and methylation. Additionally, our results illustrated that IGF2BP1 expression was positively associated with TMB in BRCA, COAD, HNSC, KICH, KIRC, KIRP, LGG, LICH, LUAD, LUSC, and SARC (**Figure 4G**, P <0.05).

Several studies have demonstrated that tumor mutation burden (TMB) and microsatellite instability (MSI) stimulate lymphocyte antitumor responses and help the immune system recognize tumors (32, 33). Our analysis revealed that IGF2BP1 expression was positively correlated with TMB in BRCA, COAD, HNSC, KICH, KIRC, KIRP, LGG, LICH, LUAD, LUSC, and SARC (**Figure 4G**, P <0.05). We also found that IGF2BP2 expression was positively associated with TMB in BRAC, HNSC, LGG, LUAD, PAAD, SKCM, STAD, and THYM (**Figure 4H**, P <0.05). Moreover, IGF2BP3 expression was significantly positively associated with TMB in ACC, BRCA, COAD, HNSC, LGG, LUAD, LUSC, READ, SARC, TGCT, and THYM (**Figure 4I**, P < 0.05). Furthermore, MSI has emerged as a key predictor of cancer immunotherapy outcome. A significant positive correlation was detected between IGF2BP1 expression and MSI in cancers such as KICH, LUSC, MESO, OV, and SARC; while, a significant negative correlation between IGF2BP1 expression and MSI was observed in COAD. Finally, we found that IGF2BP3 expression was negatively associated with MSI in DLBC and THCA (**Figures 4J–L**, P < 0.05).

### IGF2BPs are associated with TME and stemness in human pan-cancer

**Figure 5:**
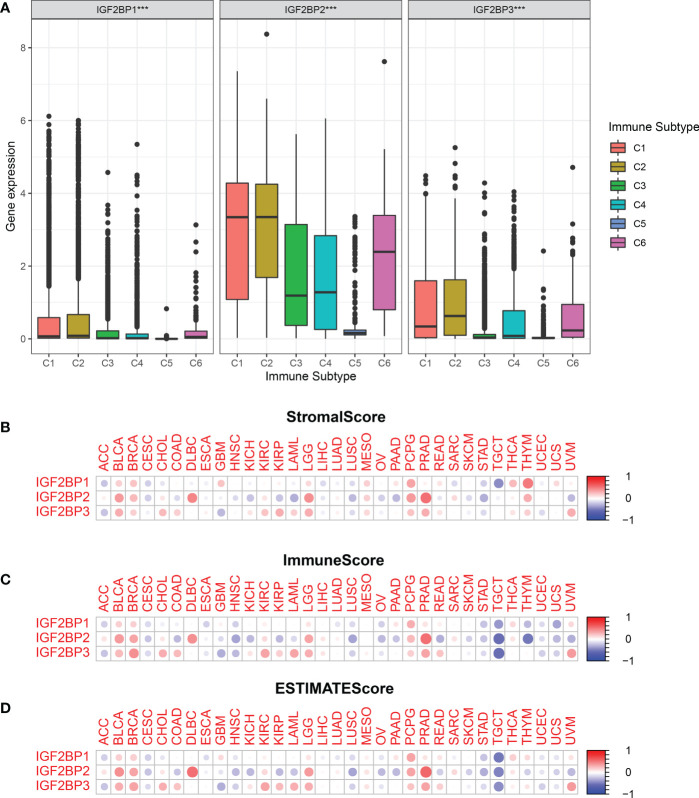
Relationship between the expression of the IGF2BPs and the tumor immune microenvironment.**(A)** Relationship between the expression of the IGF2BPs and the infiltrating immune sub-types in pan-cancer. C1: wound healing, C2: INF-r dominant, C3: inflammatory, C4: lymphocyte depleted, C5: immunologically quiet, and C6: TGFβ dominant. The correlation matrix of the IGF2BPs expression and **(B)** the stromal cells, **(C)** the immune cells, **(D)** as well as comprehensive scores of 33 cancer types based on the estimation algorithm. ***P < 0.001.

To determine how each IGF2BPs is associated with immune components, the relationship between IGF2BPs and tumor immune infiltrates were examined. Tumor immune infiltrates are classified into six subtypes: C1 (wound healing), C2 (interferon [IFN]-^γ^ dominant), C3 (inflammatory), C4 (lymphocyte-depleted), C5 (immunologically quiet), and C6 (tumor growth factor [TGF]-β dominant). We analyzed immune infiltrates in pan-cancer data from TCGA and correlated them with the level of expression of IGF2BPs.The OS across all cancer types showed that patients characterized into C3 and C5 infiltrates had better survival than that into the other four infiltrates, where patients in C4 and C6 groups had least favorable survival (Fig S3, P < 0.001). IGF2BP1-3 expression differed significantly in various tumor immune infiltrates subtypes (Fig 5A, P < 0.001). Positive correlations between higher levels of IGF2BP1-3 and C1, C2 and C6 infiltrates suggests that IGF2BPs may have a tumor promoter role, as patients belonging to those immune infiltrates subtypes had worse survival characterized with higher proliferation rate and enriched with TGFβ (**Figure 5A** and **Figure S3**). Moreover, we examined the association between the expression of IGF2BPs and immune, stromal, and ESTIMATE scores. Tumor purity is negatively correlated with stromal and immune scores. Among the different cancer types, the degree of association between IGF2BPs expression and the stromal scores varied considerably. IGF2BPs were positively associated with stromal scores in patients with BLCA, BRCA, DLBC, LGG, PCPG, and PRAD. Strikingly, IGF2BPs levels and the stromal and immune scores were significantly positively correlated in LGG (**Figures 5B–D**, P <0.05). The tumor stemness can be evaluated using RNA or DNA stemness scores, based on mRNA expression or DNA methylation patterns, respectively (34). In ACC, ESCA, LUSC, TGCT, and UCEC, IGF2BPs expression positively correlated with cancer stemness (**Figures S4A, B**).

### IGF2BPs expression is related to immune cell infiltration and immune checkpoint biomarkers in human pan-cancer

**Figure 6:**
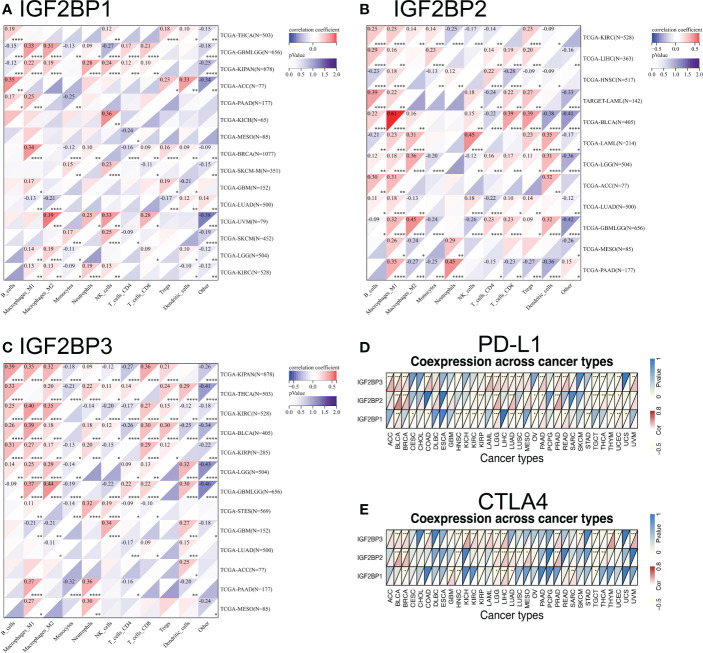
Relationship between IGF2BPs and the expression of tumor-infiltrating immune cell and immune checkpoint inhibitors. **(A-C)** Heatmap representation of the correlation between IGF2BPs expression and immune cell in pan-cancer. **(D, E)** Heatmaps represent the association of the IGF2BPs expression with PD-L1 and CTLA4. *P < 0.05, **P < 0.01, ***P < 0.001, ****P < 0.0001.

Tumor immunotherapy functions by restoring the antitumor immune response (35). We found that the expression of IGF2BPs was positively correlated in the types of cancer that were associated with prognosis based on TCGA. We investigated correlations between IGF2BPs expression and various immune cell markers, using the SangerBox database (**Figures 6A–C**). The results showed that IGF2BP3 was significantly positively correlated with macrophages, B cells, and CD8^+^ T cells in BLCA, KIRC, PAAD, and LGG (**Figure 6C**, P <0.05). Notably, IGF2BPs expression was significantly positively associated with M1 macrophages in glioma. We then investigated the correlations between IGF2BPs and over 40 common immune checkpoint genes and 22 immune cells. In various immune cell types, IGF2BPs expression was closerly associated with certain immune cell markers (**Figures S4A–F**, P <0.05). Interestingly, IGF2BP3 expression was negatively correlated with the expression of inhibitory coreceptors CTLA-4 and PD-L1 in BRCA, BLCA, LGG, LICH, PRAD, TGCT, and UVM (**Figures 6D, E**, p <0.001). Taken together, IGF2BPs expression was not only correlated with immune infiltration into the tumor, but also play a crucial role in immune evasion.

### Drug sensitivity analysis

Next, we investigated the relationship between IGF2BPs expression and the sensitivity of NCI-60 cells to chemotherapy drugs. We demonstrated the correlation between drug sensitivity and gene expression using a scatter plot based on P-values (**Figure S5C**, P <0.05). IGF2BP2 expression was negatively correlated with the sensitivity of NCI-60 cells to dexrazoxane (r = –0.547, P <0.001), AM-5992 (r=–0.520, P <0.01), SR16157 (r = –0.502, P <0.001), and etoposide (r=–0.485, P <0.001). IGF2BP3 expression was positively correlated with sensitivity to ARRY-704 (correlation coefficient =0.471, P <0.001), RO-4987655 (r =0.425, P <0.001), trametinib (r = 0.460, P <0.001), TAK-733 (r = 0.453, P <0.001), and PD-0325901 (r =0.450, P <0.001). Collectively, these results highlight the potential role of IGF2BPs in anti-cancer drug resistance.

### Potential roles of IGF2BPs in glioma tumor immunity and the TME

**Figure 7:**
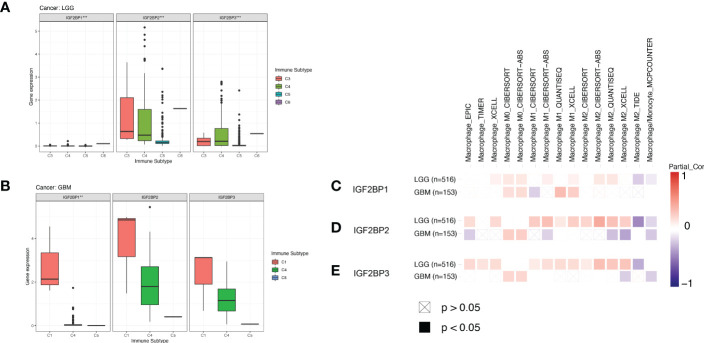
Association of IGF2BPs and tumor immune microenvironment in LGG and GBM based on TCGA. Association of the IGF2BPs expression and the immune infiltrate subtypes in **(A)** LGG and **(B)** GBM. **(C-E)** Association of the IGF2BPs expression and TAMs. Red color represents positive correlation, blue color represents negative correlation, and the deeper the color, the stronger the correlation. **P < 0.01, ***P < 0.001.

**Figure 8:**
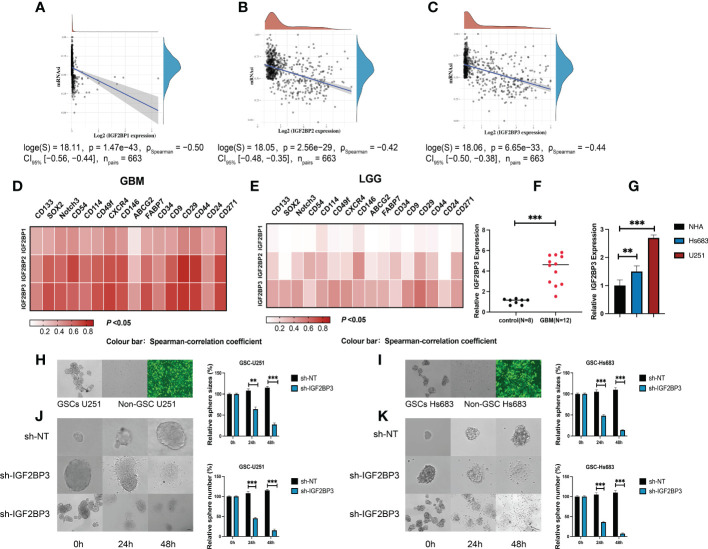
IGF2BP3 plays a key role in the maintenance and self-renewal of GSCs. **(A–C)** The correlation of stemness score and IGF2BPs gene expression. **(D, E)** GEPIA database showing IGF2BPs expression correlation with stem gene in high-grade glioma tissues compared with that in low-grade glioma tissues. **(F)** The serum level of IGF2BP3 was significantly increased in the GBM patients. **(G)** RT-qPCR analysis of IGF2BP3 expression in glioma cell lines (U251 and Hs 683). **(H, I)** The representative images of 2 established neutrosphere-cultured GSCs and lentiviral transfection efficiency. **(J, K)** Sphere formation of U251and HS683 after infection with shRNA-IGF2BP3 and shRNA-NT was evaluated by the sphere formation assay. Bar: 100μm. **P < 0.01, ***P < 0.001.

We next compared the expression levels of IGF2BPs across the immune subtypes in LGG and GBM, using TCGA RNAseq data. We found that the expression of IGF2BPs was significantly different across the C3, C4, C5, and C6 immune subtypes in LGG (**Figure 7A**, P<0.05). IGF2BP1 expression was significantly different between C1, C4 and C5 immune subtypes in GBM; however, the same was not observed for IGF2BP2 and GF2BP3 (**Figure 7B**; P < 0.05). Next, using the TIMER2 database, we found that IGF2BPs expression was positively correlated with the infiltration of macrophages in LGG and GBM (**Figures 7C–E**). To assess the relevance of IGF2BPs expression to cancer stemness, we analyzed mRNA expression-based stemness index (mRNAsi) scores, which demonstrated that the mRNAsi score was significantly higher in glioma tissues(**Figures 8A-C**, P <0.05). We then proceeded to evaluate the association between IGF2BPs and common stem cell markers expressed in glioma tissues of different grades. We found that IGF2BP1–3 expression was more highly associated with common stem cell markers expressed in GBM than in LGG tissues (**Figures 8D, E**). This suggests that IGF2BPs expression is positively correlated with glioma stemness levels and grades. Interestingly, of the three IGF2BPs, IGF2BP3 displayed the highest correlation with stem cell markers.

### IGF2BP3 is associated with glioma stemness

To further validate the function of IGF2BP3 in the regulation of glioma stemness, we verified the expression of IGF2BP3 in glioma cells (U251 and HS683) and in the peripheral blood of GBM patients. The qRT-PCR results show that the expression level of IGF2BP3 in blood of GBM patients was significantly higher than that of normal controls (**Figure 8F**, P <0.05). Similarly, the IGF2BP3 was more highly expressed in the GBM cell lines (U251 and HS683) than in normal human astrocyte cells (NHA) (**Figure 8G**, P <0.05). We then went on to elucidate the role of IGF2BP3 in U251 and HS683 cells *in vitro*. We depleted IGF2BP3 from U251 and HS683 cells by introducing IGF2BP3-specific shRNA sequences using lentiviral vectors. Fluorescence microscopy showed that the cells were successfully (nearly 100%; MOI = 10) transduced 72 h after lentiviral infection (**Figures 8H, I**). The results show that lentiviral transduction with sh-IGF2BP3 reduced the stemness, and the sphere size and number were also suppressed by IGF2BP3 knockdown in GSC-U251 and GSC-Hs683 cells (**Figures 8J, K**, P <0.05). These findings suggested that IGF2BP3 may be critical for the maintenance of GSCs characteristics.

### Inhibition of proliferation, migration, and invasion in GSCs and glioma cells following IGF2BP3 knockdown

**Figure 9:**
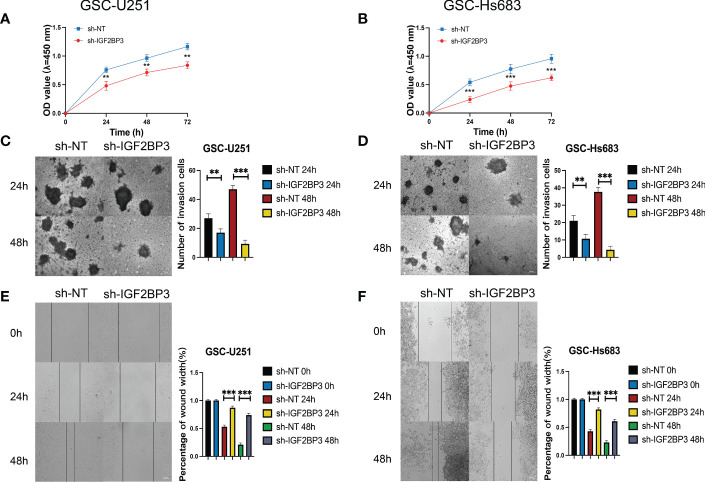
IGF2BP3 knockdown inhibits the cell proliferation, migration, and invasion of GSCs. **(A, B)** Cell proliferation ability of U251 and HS683 cells transfected with sh-IGF2BP3 was evaluated by CCK8 assay. **(C, D)** Cell migration capability of U251 and HS683 cells transfected with sh-IGF2BP3 was evaluated by wound healing assays. **(E, F)** The influence on cell migration and invasion abilities of U251 and HS683 cells transfected with sh-IGF2BP3 was assessed by transwell migration invasion assays. Bar: 100μm. **P < 0.01, ***P < 0.001.

**Figure 10:**
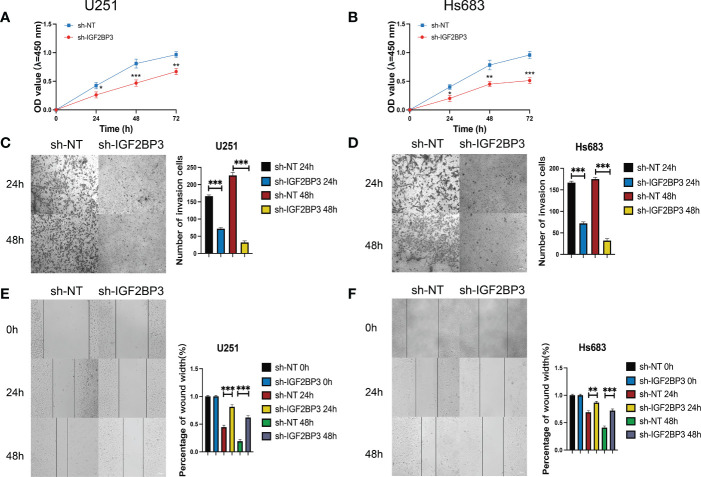
IGF2BP3 knockdown impairs glioma cell proliferation, migration, and invasion. **(A, B)** Cell proliferation ability of U251 and HS683 cells transfected with sh-IGF2BP3 was evaluated by CCK8 assay. **(C, D)** Cell migration capability of U251 and HS683 cells transfected with sh-IGF2BP3 was evaluated by wound healing assays. **(E, F)** The in-fluence on cell migration and invasion abilities of U251 and HS683 cells transfected with sh-IGF2BP3 was assessed by transwell migration invasion assays. Bar: 100μm. *P < 0.05, **P < 0.01, ***P < 0.001.

We then performed the CCK-8 cell proliferation assay to determine whether IGF2BP3 regulates the proliferation of GSCs, and glioma cell lines. The GSC-U251 and GSC-HS683 cells showed a markedly decreased rate of proliferation, compared with control cells, after being transduced with sh-IGF2BP3 (**Figures 9A, B**, P <0.05). Moreover, knockdown of IGF2BP3 decreased the migration of U251-GSC and HS683-GSC cells in a Transwell migration assay (**Figures 9C, D**, P <0.05). Lowering IGF2BP3 expression also significantly reduced the invasion capacity of GSC-U251 and GSC-HS683 cells in a wound healing assay (**Figures 9E, F**, P <0.05).Similarly, IGF2BP3 knockdown significantly reduced U251 and HS683 cell proliferation compared with the negative control (**Figures 10A, B**, P <0.05). Furthermore, knockdown of IGF2BP3 led to the reduced migration (**Figures 10C, D**, P <0.05) and invasion (**Figures 10E, F**, P <0.05) of the U251 and HS683 cells. Thus, IGF2BP3 may play a significant role in the growth and development of glioma stem cells.

## Discussion

It is well established that m6A regulates gene expression, thereby controlling processes ranging from cell self-renewal to differentiation, invasion, and apoptosis. Alterations in m6A levels affect cancer development and progression by regulating the expression of immune and stem cell genes (36, 37). IGF2BPs are m6A readers, whose role in cancer onset and progression has been documented in a variety of human malignancies. For instance, IGF2BP1 promotes HCC cell proliferation by binding to and stabilizing the c-Myc and Ki-67 proteins, two important pro-tumorigenic factors in HCC (38). In addition, IGF2BP3 has been demonstrated to enhance the tumor growth and metastatic spread of colorectal cancer cells (39). In this study, IGF2BP1 and 3 are either absent or expressed at very low levels in most normal tissues and cancer cell lines. However, IGF2BPs can be re-expressed upon malignant transformation and are found in a broad range of cancer types where their expression often correlates with poor prognosis. We performed a pan-cancer expression analysis of IGF2BP1–3 and found that the genes encoding these proteins were significantly upregulated in most of the tumor tissues, compared with adjacent normal tissues. We further validated the expression of IGF2BP2 and IGF2BP3 in tumor tissues (COAD, ESCA, and STAD) using qRT-PCR and immunofluorescence staining. The results of these analyses were consistent with those of the bioinformatics analysis. Subsequently, Kaplan–Meier survival analysis revealed that IGF2BPs overexpression was correlated with the poor OS of cancer patients, and especially those with glioma. These observations led us to speculate that aberrant IGF2BP2 and IGF2BP3 expression might regulate cancer progression and may act as a therapeutic target for cancers.

The proper function of IGF2BPs is essential to an effective antitumor response. Tumor cells have devised numerous ways to escape the immune system. A number of these strategies involve alterations of IGF2BPs genes. In this study, we have comprehensively investigated the relationship between IGF2BPs gene expression and the TME and stemness of various types of cancer. MMR contributes to maintaining genome stability. Thus, the loss of IGF2BPs function leads to DNA replication errors occur, eventually increasing the frequency of somatic mutations. Genomic DNA methylation is epigenetic modification that regulates gene expression. All cancers exhibit mutations in their DNA methylation machinery, which play a critical role in tumor development and progression. In the present study, we demonstrated that IGF2BPs expression correlated with DNA methylation levels and MMRs patterns in different types of cancer, suggesting that IGF2BPs may play a role in tumor progression by mediating DNA repair and methylation. Meanwhile, the main reason for cancer formation is genetic mutations (40). Therefore, the presence of certain genetic mutations can be used to predict prognosis and treatment response in patients (41–43). TMB and MSI are very common in multiple cancer subtypes and may have a profound effect on patient survival (44). It has been shown that higher TMB correlates with a better response to immune checkpoint inhibitors and longer OS (33). In this study, a correlation was observed between IGF2BPs expression and the TMB and MSI of some types of cancer. Moreover, we found a significant positive correlation between IGF2BPs mRNA levels and the TMB and MSI in ACC, BLCA, BRCA, CESC, LUAD, LUSC, PRAD, SARC, STAD, and UCEC; however, a negative correlation was found in THYM. Therefore, overall, IGF2BPs expression was positively correlated with the TMB and MSI, meaning that IGF2BPs could be used as potential predictive marker for the efficacy of immunotherapy. However, further experiments are needed to confirm these findings.

Cancer research has become increasingly focused on the anti-tumor immune response (45). A previous study suggested that absence of IGF2BPs enhanced the ability of dendritic cells (DCs) to cross-present antigens and prime CD8^+^ T cells responses (5). Meanwhile, other researchers have shown that IGF2BP3 inhibits CD8^+^ T cell responses to facilitate tumor immune evasion by promoting the deubiquitination of PD-L1 in non-small cell lung cancer (46). We found that the expression of IGF2BP1–3 was mostly positively correlated with more aggressive subtypes of immune infiltrates (i.e., C1, C2, and C6). Given the essential roles of immune checkpoints in diverse cancers, we further analyzed the correlation between IGF2BPs expression and tumor immunity. We found that IGF2BPs expression was positively correlated with over 40 immune checkpoint genes in various cancers, including BRCA, GBM, HNSC, LGG, SARC, THCA, and UVM. Moreover, IGF2BPs expression was significantly positively correlated with tumor infiltrations of macrophages, B cells, and CD8^+^ T cells in BLCA, KIRC, PAAD, and LGG. Additionally, previous studies have shown that IGF2BPs influence tumor growth and immune responses within TME-associated macrophages (47). Therefore, it is possible that IGF2BPs are involved in macrophage polarization towards the M1 subset and the subsequent activation of the immunosuppressive response. Specifically, we compared the expression levels of IGF2BPs across the immune subtypes in LGG and GBM. IGF2BP1 expression was significantly different in the C1, C4, and C5 immune subtypes in GBM; however, the expression levels of IGF2BP2 and IGF2BP3 were similar. The above results suggest that IGF2BPs might be involved in tumor immune escape during cancer immunotherapy. Notably, we found that the expression of IGF2BPs was differentially associated with the immune response in patients with different grades of glioma. For instance, IGF2BP3 expression was significantly positively correlated with LGG, while no correlation with GBM was observed. This may also imply a novel tumor immune escape mechanism. Thus, based on the findings presented in this manuscript, IGF2BPs may promote or inhibit cancer progression by recruiting and regulating infiltrating immune cells and play a critical role in cancer immunity.

CSCs have been linked to a wide range of cancers and play a role in cancer recurrence and drug resistance (48). But the mechanism by which GSCs migrate to and invade tissues are not fully understood. Several studies have reported that IGF2BPs provides a link between stem cell maintenance in normal development and cancer (49). However, knowledge regarding the involvement of IGF2BPs in tumor immunity and stemness maintenance in cancer is still lacking. A recent study found that the regulation of SLUG by IGF2BP3 plays a crucial role in maintaining the stem cell properties of breast cancer cells (50). We found that IGF2BP3 knockdown was able to reduce GSC stemness and proliferation as well as promote GSC differentiation and apoptosis. Additionally, IGF2BP3 knockdown significantly decreased the proliferation of glioma cells and promoted their apoptosis. Thus, IGF2BP3 may act as a potent adjuvant for gene-targeted therapy in glioma. It is likely that these findings are just a small fraction of what can be learned about IGF2BPs function in CSCs; the underlying mechanisms and pathways involved are yet to be delineated. Thus, further studies focusing on the regulatory mechanisms of IGF2BP3 in glioma could further our understanding of glioma carcinogenesis.

There are several limitations to the present study. First, the use of microarray datasets from different public datasets has inevitably introduced systematic bias. Second, the functional experiments were performed in *in vitro* cultured cells. Third, despite concluding that a close association exists between IGF2BPs expression, immune cell infiltration, and cancer prognosis, we obtained no evidence showing that IGF2BPs influence prognosis by directly taking participating in immune infiltration. However, there are no robust studies demonstrating that IGF2BP-targeting drugs inhibit tumor growth or improve survival. Therefore, future research should evaluate the role of IGF2BPs in cancer immune infiltration as well as the development and testing of antitumor immunotherapies targeting IGF2BPs.

## Conclusion

In this study, we performed a preliminary evaluation of the prognostic value of IGF2BPs expression in pan-cancer, using bioinformatics prediction and experimental validation approaches. Overall, our study highlights the important role of IGF2BPs in multiple cancer types. Specifically, we demonstrated the role of IGF2BP3 as a potential negative regulator in glioma, which it performs by modulating the TME and stemness. These findings will broaden our understanding of the role of IGF2BPs in cancer and potentially inform the development of targeted immunotherapies.

## Data availability statement

Publicly available datasets were analyzed in this study. This data can be found here: The original contributions presented in the study are included in the article/**Supplementary Material**. Further inquiries can be directed to the corresponding authors.

## Ethics statement

The studies involving human participants were reviewed and approved by ethics committee of the Nanyang Central Hospital (Nanyang, Henan, P.R. China). The patients/participants provided their written informed consent to participate in this study.

## Author contributions

QX, FD, and YK conceived and designed the study. WS and SZ collected the data. HZ and WS analyzed and interpreted the data. QX, HZ, and QD verified the data. YG and KH performed all the statistical analysis. QX, FD, WS, and HZ prepared figures and prepared the manuscript. All authors have read and approved the final version. All authors contributed to the article and approved the submitted version.
