# Supplementary material for: A pan-cancer landscape of IGF2BPs and their association with prognosis, stemness and tumor immune microenvironment
Source: Front Oncol. 2023 Jan 4;12:1049183. doi: 10.3389/fonc.2022.1049183 (PMC9846525; doi:10.3389/fonc.2022.1049183)
Supplement: Supplementary file 1 [file DataSheet_1.docx]

Supplementary Material

## Supplementary Figures


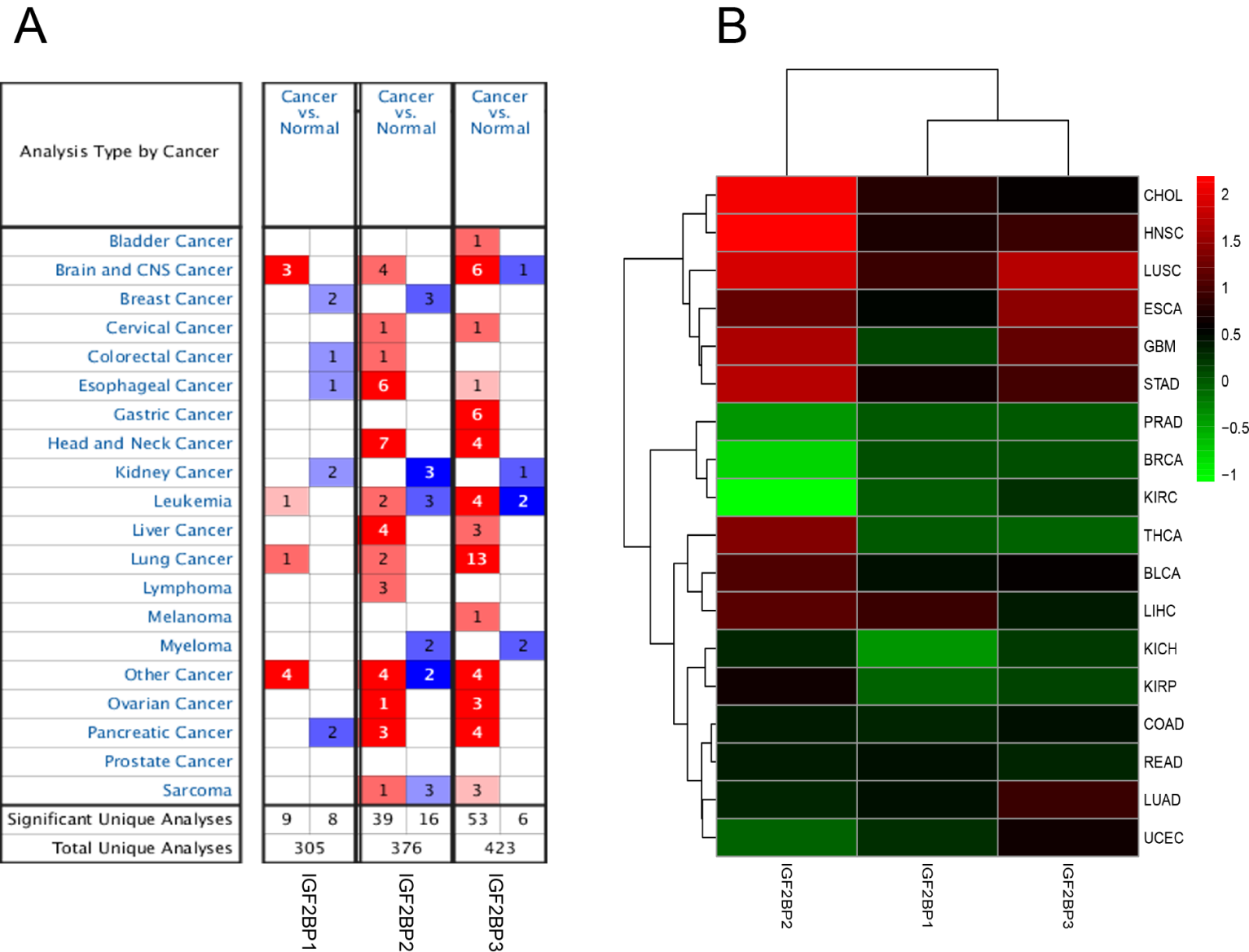
**Supplementary Figure 1.**The expression levels of IGF2BPs in different types of human cancers. (A) Transcript levels of IGF2BPs in different cancers based on the ONCOMINE database. (B) Heatmap showing the difference in the IGF2BPs expression between the primary tumor and the adjacent normal tissues, which was based on log2 (fold change) of 16 cancer types that had more than five adjacent normal samples.


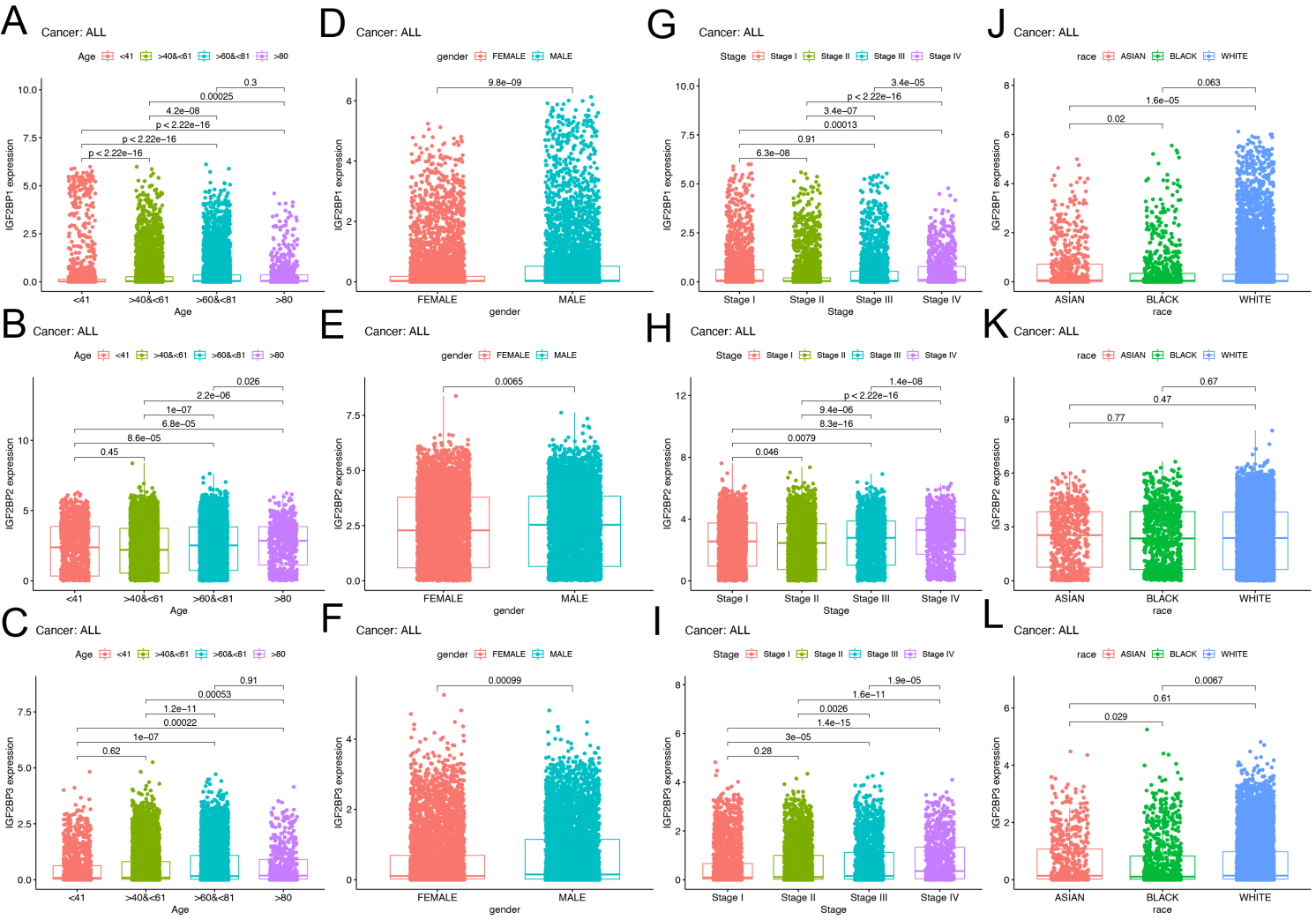


**Supplementary Figure 2**: Relationship between IGF2BPs expression and clinical features in pan-cancer base on TCGA. Box-plots indicate IGF2BPs expression in different clinical features, including (A-C) age, (D-F) gender, (G-I) stage, and (J-L) race of pan-cancer from the TCGA dataset. *P < 0.05, **P < 0.01, ***P < 0.001.

**Supplementary Figure 3**: Kaplan-Meier survival curve to show the overall survival difference among the six immune subtypes across all cancer types.


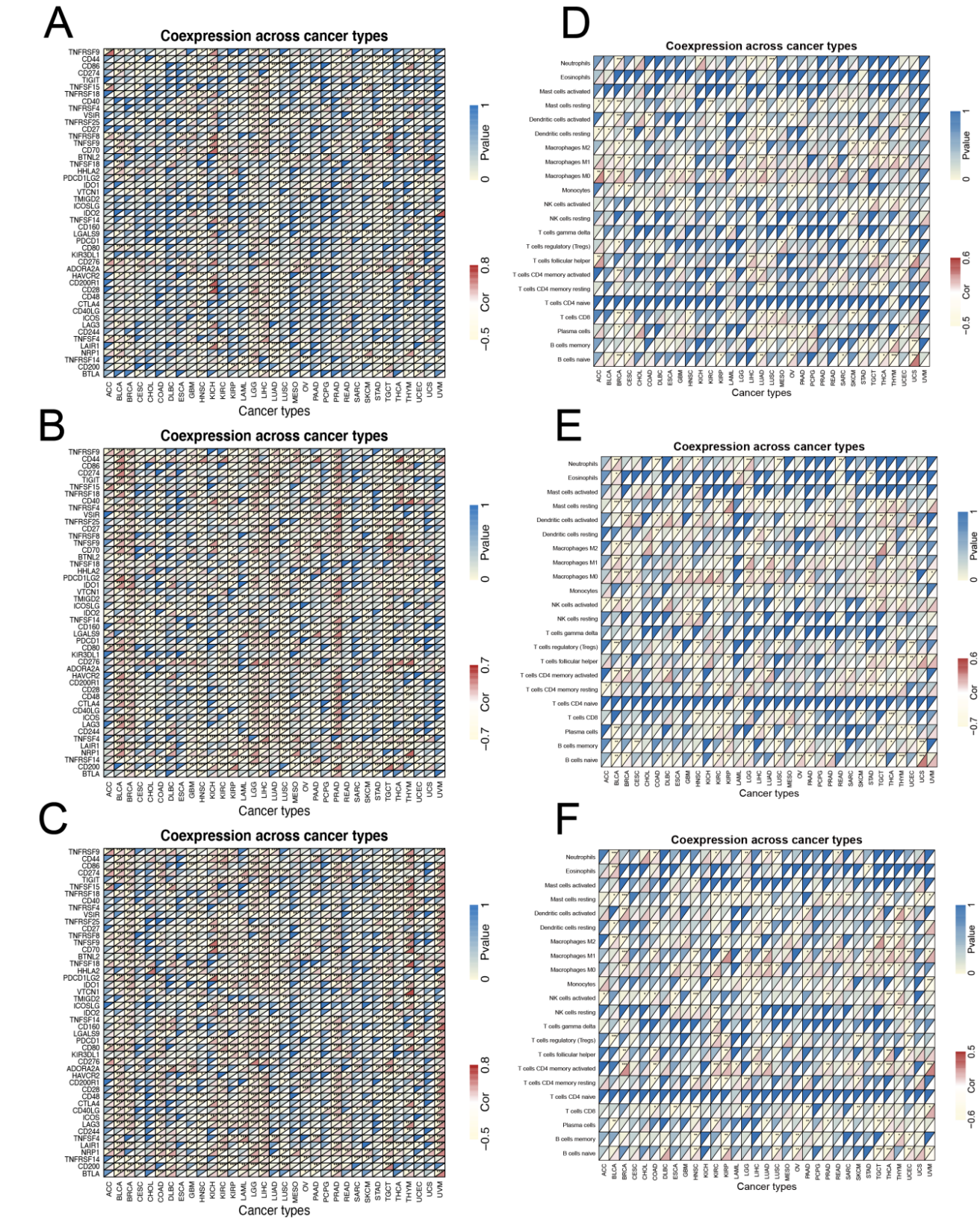


**Supplementary Figure 4:** Association of IGF2BPs expression with immune checkpoint markers expression and immune cells in pan-cancer. (A-C) Heat map shown the association of (A) IGF2BP1, (B) IGF2BP2, and (C) IGF2BP3 expression with immune checkpoint markers. (D-F) Heatmap shown the association of (D) IGF2BP1, (E) IGF2BP2, and (F) IGF2BP3 expression with immune cells. For each pair, the top right triangle is colored to represent the P-value, and the bottom left triangle is colored to represent the correlation coefficient. *P < 0.05, **P < 0.01, ***P < 0.001.


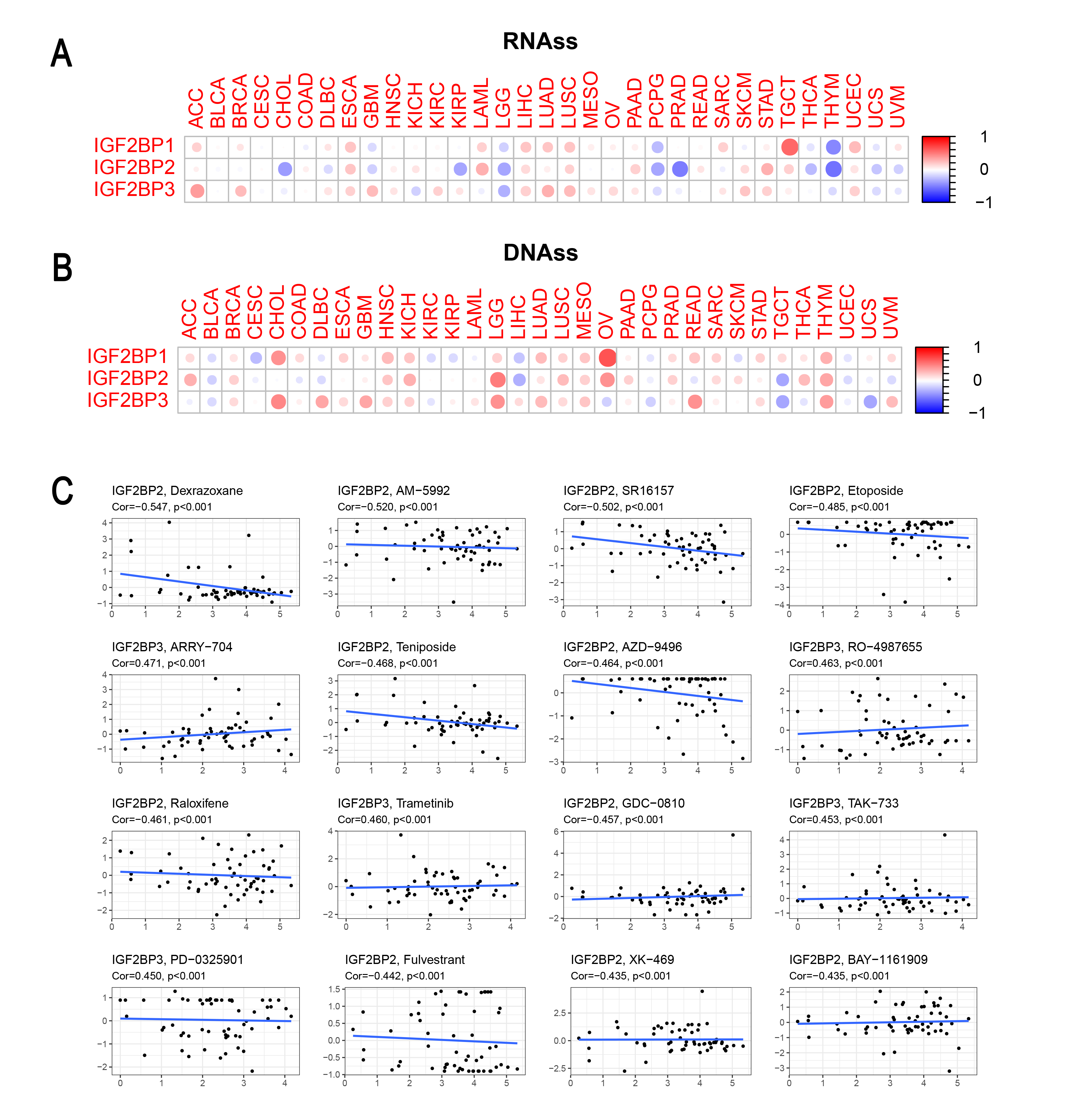


**Supplementary Figure 5**: Relationship between the IGF2BPs expression levels and the tumor stemness. Correlation matrix of the IGF2BPs expression and the cancer RNA and DNA stemness scores, (A) RNAss and (B) DNAss, respectively. (C)Association of IGF2BPs expression with drug responses.

**Supplementary Table 1**: Clinical characteristics of ESCA, STAD, and COAD patients.

| **Clinical characteristics** | **ESCA tissues (n=10)** | **STAD tissues (n=10)** | **COAD tissues (n=10)** |
| --- | --- | --- | --- |
| **Gender** |  |  |  |
| Male | 9 | 8 | 6 |
| Female | 1 | 2 | 4 |
| **Age** |  |  |  |
| <60 | 2 | 7 | 4 |
| ≥60 | 8 | 3 | 6 |
| **Pathologic grade** |  |  |  |
| I+II | 6 | 10 | 9 |
| III+IV | 4 | 0 | 1 |
| **Lymphatic invasion** |  |  |  |
| No | 6 | 7 | 8 |
| Yes | 4 | 3 | 2 |
| **Lymph node metastasis** |  |  |  |
| No | 3 | 2 | 2 |
| Yes | 7 | 8 | 8 |
| **T stage** |  |  |  |
| T1 | 0 | 1 | 0 |
| T2 | 2 | 2 | 1 |
| T3 | 7 | 3 | 8 |
| T4 | 1 | 4 | 1 |
| **N stage** |  |  |  |
| N0 | 4 | 7 | 5 |
| N1 | 3 | 1 | 2 |
| N2 | 3 | 1 | 2 |
| N3 | 0 | 0 | 1 |
| **M stage** |  |  |  |
| M0 | 10 | 8 | 9 |
| M1 | 0 | 2 | 1 |

**Supplementary Table 2**: Clinical characteristics of the GBM, ESCA, STAD, and COAD patients.

| **Clinical characteristics** | **GBM blood (n=12)** | **ESCA tissues (n=3)** | **STAD tissues (n=3)** | **COAD tissues**  **(n=3)** |
| --- | --- | --- | --- | --- |
| **Gender** |  |  |  |  |
| Male | 7 | 3 | 2 | 3 |
| Female | 5 | 0 | 1 | 0 |
| **Age** |  |  |  |  |
| <60 | 4 | 2 | 2 | 0 |
| ≥60 | 9 | 1 | 1 | 3 |
| **Pathologic grade** |  |  |  |  |
| I+II | 0 | 3 | 3 | 2 |
| III+IV | 12 | 0 | 0 | 1 |
| **Lymphatic invasion** |  |  |  |  |
| No | 6 | 2 | 3 | 2 |
| Yes | 6 | 1 | 0 | 1 |
| **Lymph node metastasis** |  |  |  |  |
| No | 3 | 1 | 2 | 2 |
| Yes | 7 | 2 | 1 | 1 |
| **T stage** |  |  |  |  |
| T1 | 0 | 0 | 1 | 0 |
| T2 | 3 | 2 | 2 | 1 |
| T3 | 7 | 1 | 0 | 1 |
| T4 | 2 | 0 | 0 | 1 |
| **N stage** |  |  |  |  |
| N0 | 4 | 0 | 3 | 0 |
| N1 | 5 | 3 | 0 | 2 |
| N2 | 3 | 0 | 0 | 0 |
| N3 | 0 | 0 | 0 | 1 |
| **M stage** |  |  |  |  |
| M0 | 12 | 3 | 3 | 2 |
| M1 | 0 | 0 | 0 | 1 |
