# Supplementary material for: A pan-cancer landscape of IGF2BPs and their association with prognosis, stemness and tumor immune microenvironment
Source: Front Oncol. 2023 Jan 4;12:1049183. doi: 10.3389/fonc.2022.1049183 (PMC9846525; doi:10.3389/fonc.2022.1049183)
Supplement: Supplementary file 3 [file DataSheet_3.zip › Σ╗úτáü/IGF2BP1 stage.zip!/IGF2BP1 /ALLstage/Stage.ALL.pdf]

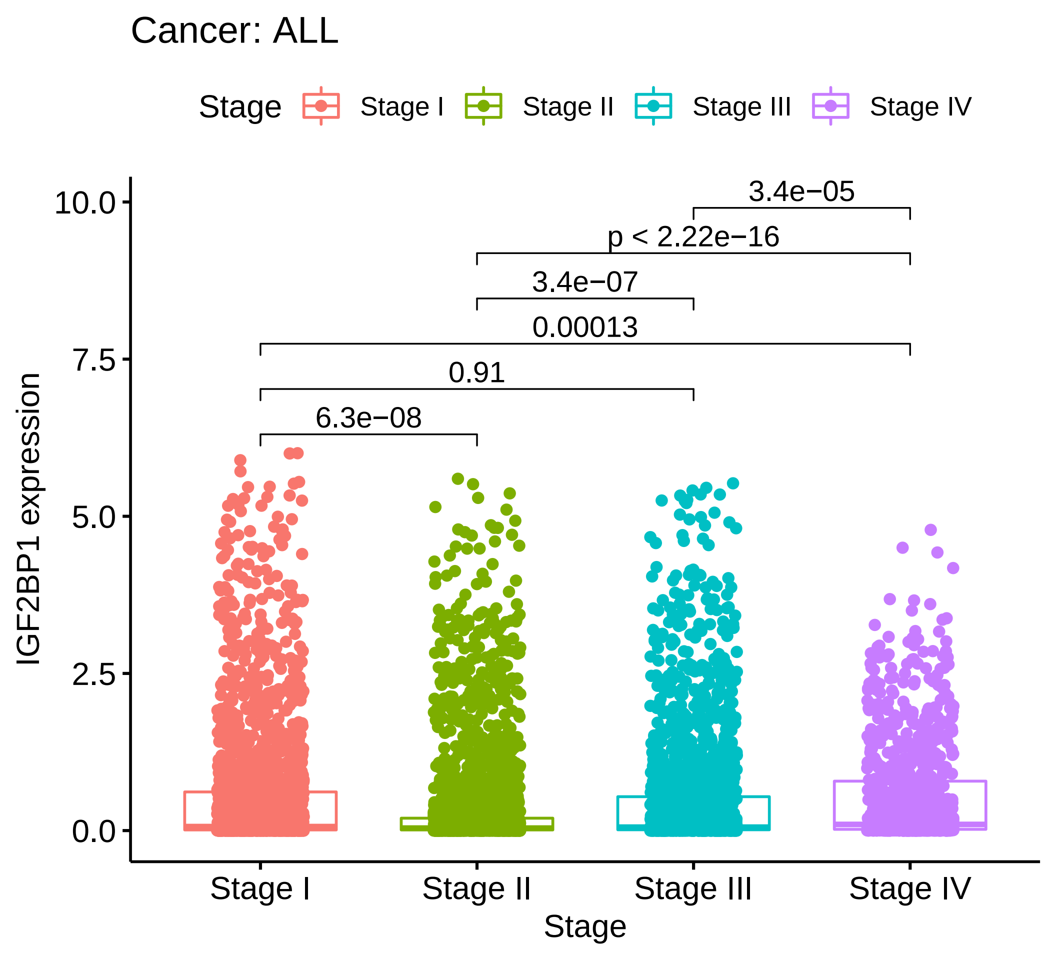

Cancer: ALL
Stage
Stage I
Stage II
Stage III
Stage IV
IGF2BP1 expression
10.0
7.5
5.0
2.5
0.0
3.4e−05
p < 2.22e−16
3.4e−07
0.00013
0.91
6.3e−08
Stage I
Stage II
Stage III
Stage IV
Stage
