# Supplementary material for: A pan-cancer landscape of IGF2BPs and their association with prognosis, stemness and tumor immune microenvironment
Source: Front Oncol. 2023 Jan 4;12:1049183. doi: 10.3389/fonc.2022.1049183 (PMC9846525; doi:10.3389/fonc.2022.1049183)
Supplement: Supplementary file 3 [file DataSheet_3.zip › Σ╗úτáü/IGF2BP1 stage.zip!/IGF2BP2/ALLAGE/Age.ALL.pdf]

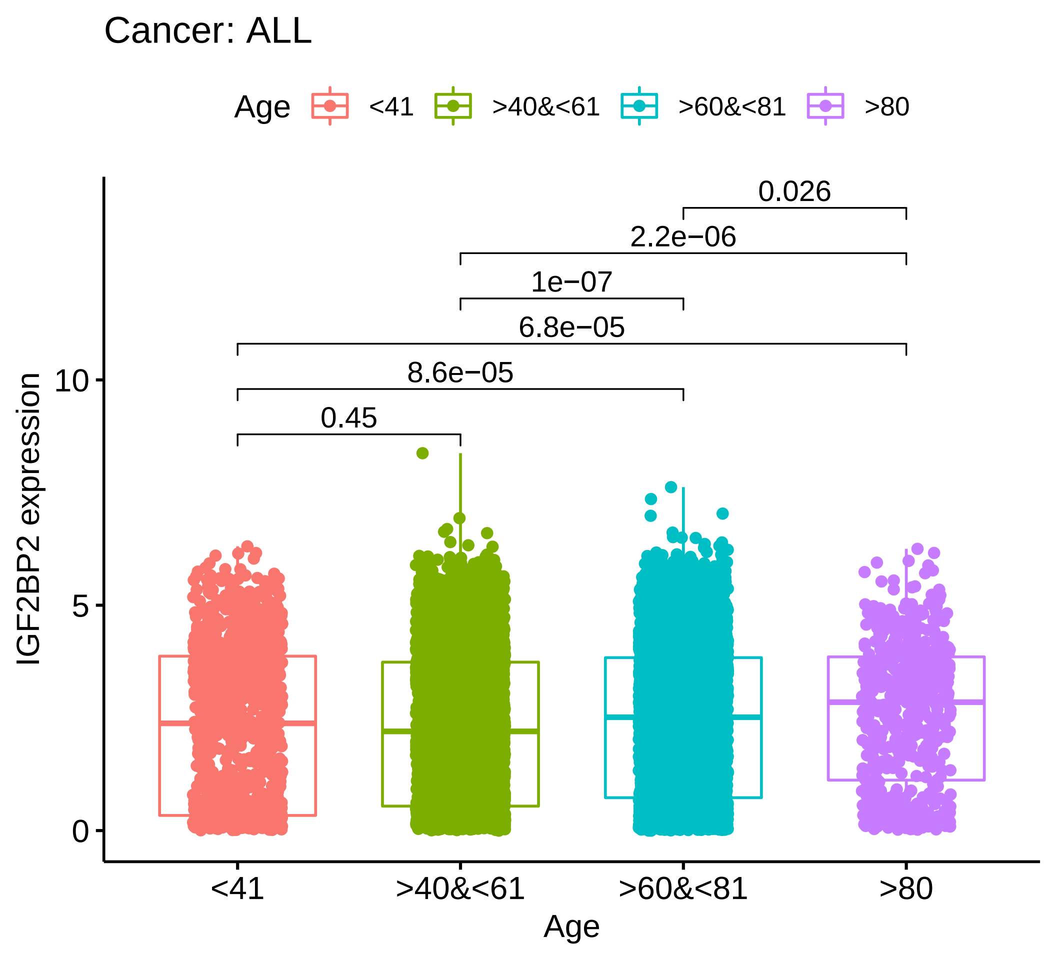

Cancer: ALL
Age
<41
>40&<61
>60&<81
>80
0.026
2.2e−06
1e−07
6.8e−05
8.6e−05
0.45
IGF2BP2 expression
10
5
0
<41
>40&<61
>60&<81
>80
Age
