# Supplementary material for: A pan-cancer landscape of IGF2BPs and their association with prognosis, stemness and tumor immune microenvironment
Source: Front Oncol. 2023 Jan 4;12:1049183. doi: 10.3389/fonc.2022.1049183 (PMC9846525; doi:10.3389/fonc.2022.1049183)
Supplement: Supplementary file 3 [file DataSheet_3.zip › Σ╗úτáü/IGF2BP1 stage.zip!/IGF2BP2/ALLRACE/race.ALL.pdf]

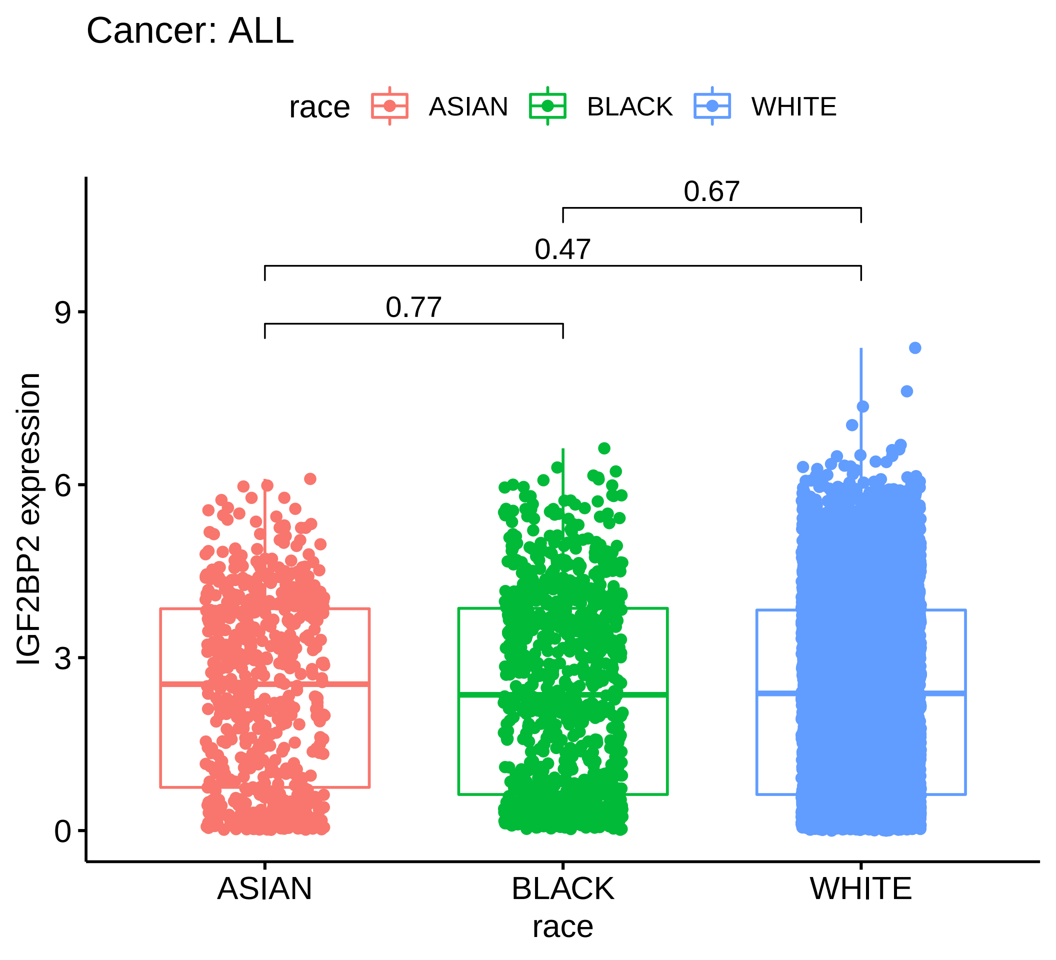
Cancer: ALL
race
ASIAN
BLACK
WHITE
0.67
0.47
0.77
IGF2BP2 expression
9
6
3
0
ASIAN
BLACK
WHITE
race
