# Supplementary material for: A pan-cancer landscape of IGF2BPs and their association with prognosis, stemness and tumor immune microenvironment
Source: Front Oncol. 2023 Jan 4;12:1049183. doi: 10.3389/fonc.2022.1049183 (PMC9846525; doi:10.3389/fonc.2022.1049183)
Supplement: Supplementary file 3 [file DataSheet_3.zip › Σ╗úτáü/IGF2BP1 stage.zip!/IGF2BP3/ALLstage/Stage.ALL.pdf]

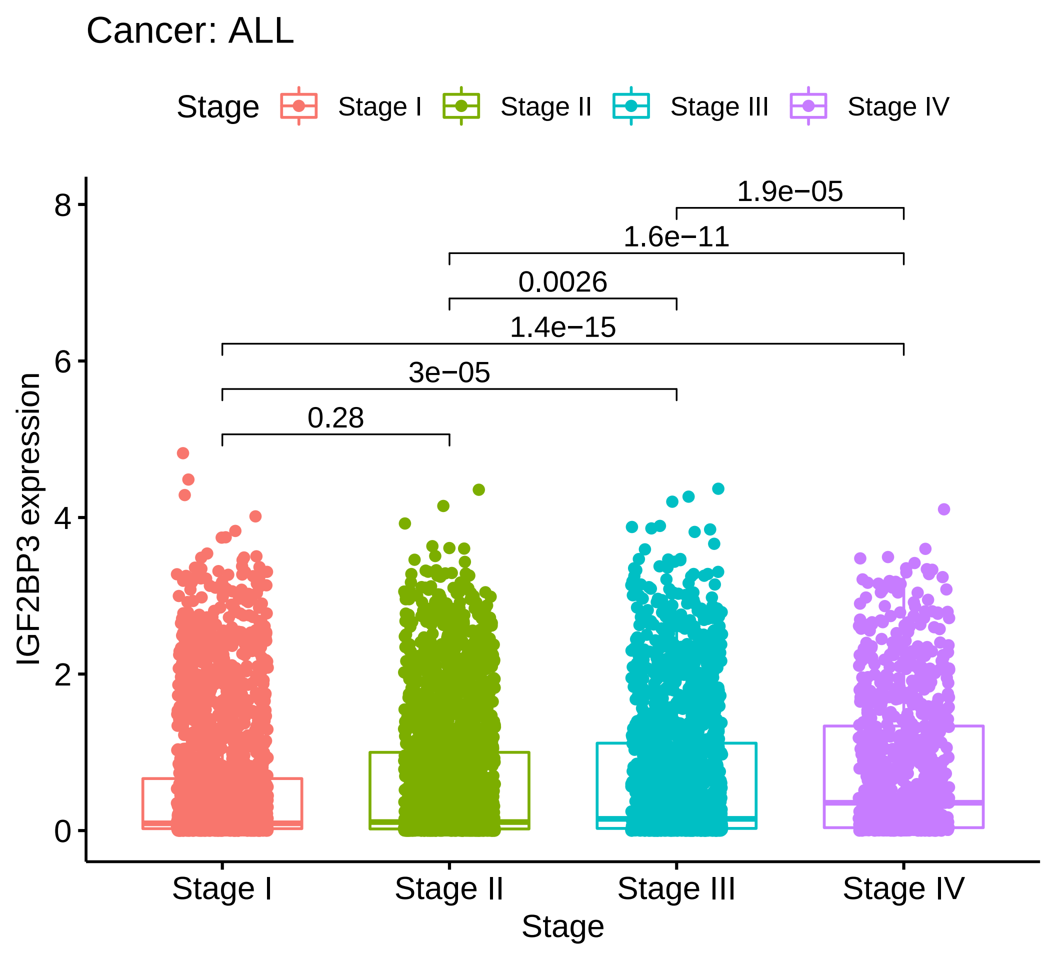

Cancer: ALL
Stage
Stage I
Stage II
Stage III
Stage IV
1.9e−05
1.6e−11
0.0026
1.4e−15
3e−05
0.28
IGF2BP3 expression
8
6
4
2
0
Stage I
Stage II
Stage III
Stage IV
Stage
