# Supplementary material for: A pan-cancer landscape of IGF2BPs and their association with prognosis, stemness and tumor immune microenvironment
Source: Front Oncol. 2023 Jan 4;12:1049183. doi: 10.3389/fonc.2022.1049183 (PMC9846525; doi:10.3389/fonc.2022.1049183)
Supplement: Supplementary file 3 [file DataSheet_3.zip › Σ╗úτáü/IGF2BP1 stage.zip!/IGF2BP3/ALLgender/gender.ALL.pdf]

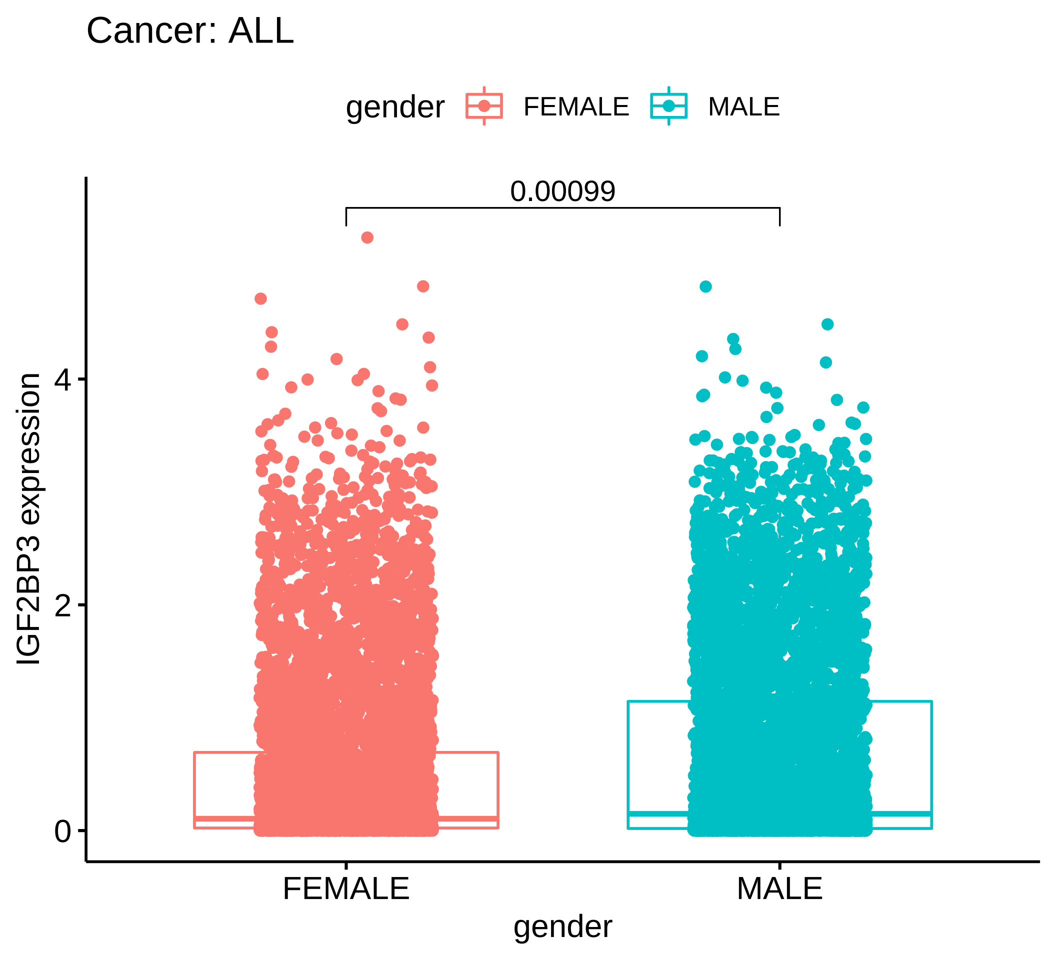
Cancer: ALL
gender
FEMALE
MALE
0.00099
IGF2BP3 expression
4
2
0
FEMALE
MALE
gender
