# Supplementary material for: A pan-cancer landscape of IGF2BPs and their association with prognosis, stemness and tumor immune microenvironment
Source: Front Oncol. 2023 Jan 4;12:1049183. doi: 10.3389/fonc.2022.1049183 (PMC9846525; doi:10.3389/fonc.2022.1049183)
Supplement: Supplementary file 3 [file DataSheet_3.zip › Σ╗úτáü/IGF2BPs heatmap.zip!/IGF2BP1 MSIradar/msiradar.pdf]

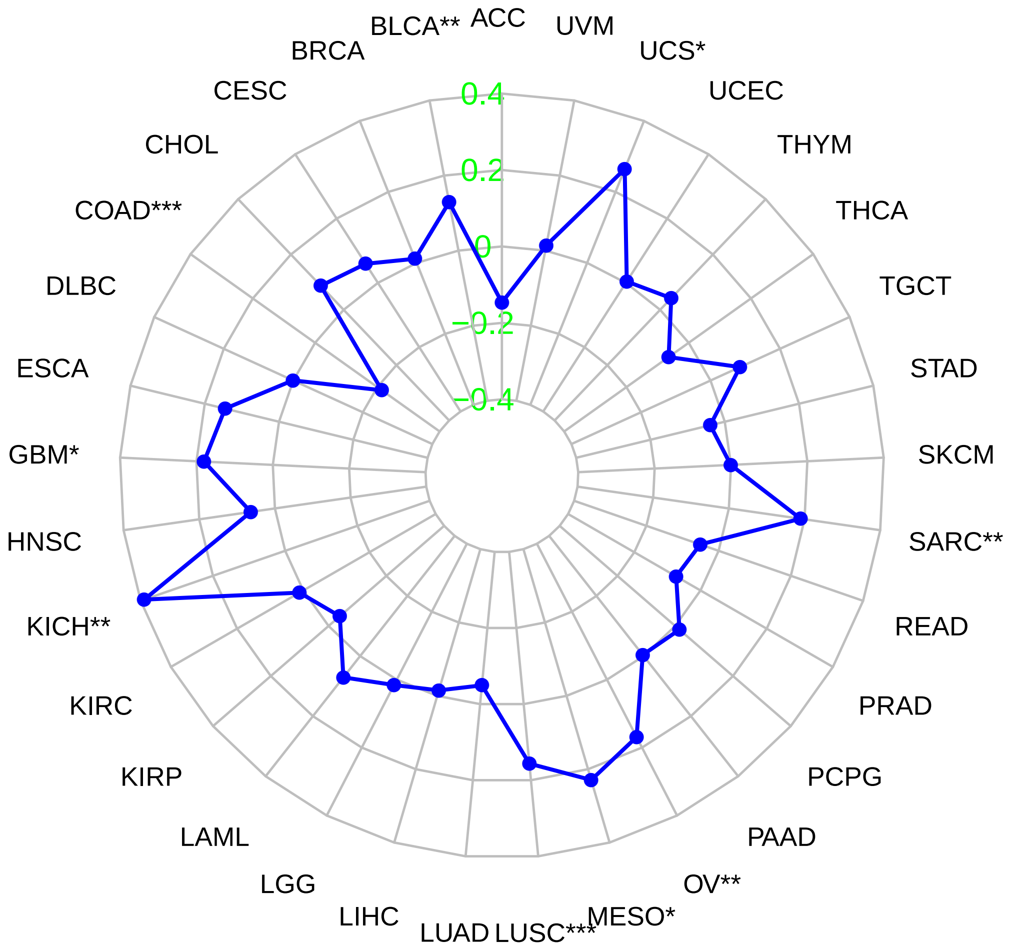
ACC
UVM
UCS*
UCEC
THYM
THCA
TGCT
STAD
SKCM
SARC**
READ
PRAD
PCPG
PAAD
OV**
MESO*
LUSC***
LUAD
LIHC
LGG
LAML
KIRP
KIRC
KICH**
HNSC
GBM*
ESCA
DLBC
COAD***
CHOL
CESC
BRCA
BLCA**
0.4
0.2
0
−0.2
−0.4
