# Supplementary material for: A pan-cancer landscape of IGF2BPs and their association with prognosis, stemness and tumor immune microenvironment
Source: Front Oncol. 2023 Jan 4;12:1049183. doi: 10.3389/fonc.2022.1049183 (PMC9846525; doi:10.3389/fonc.2022.1049183)

Coexpression across cancer types

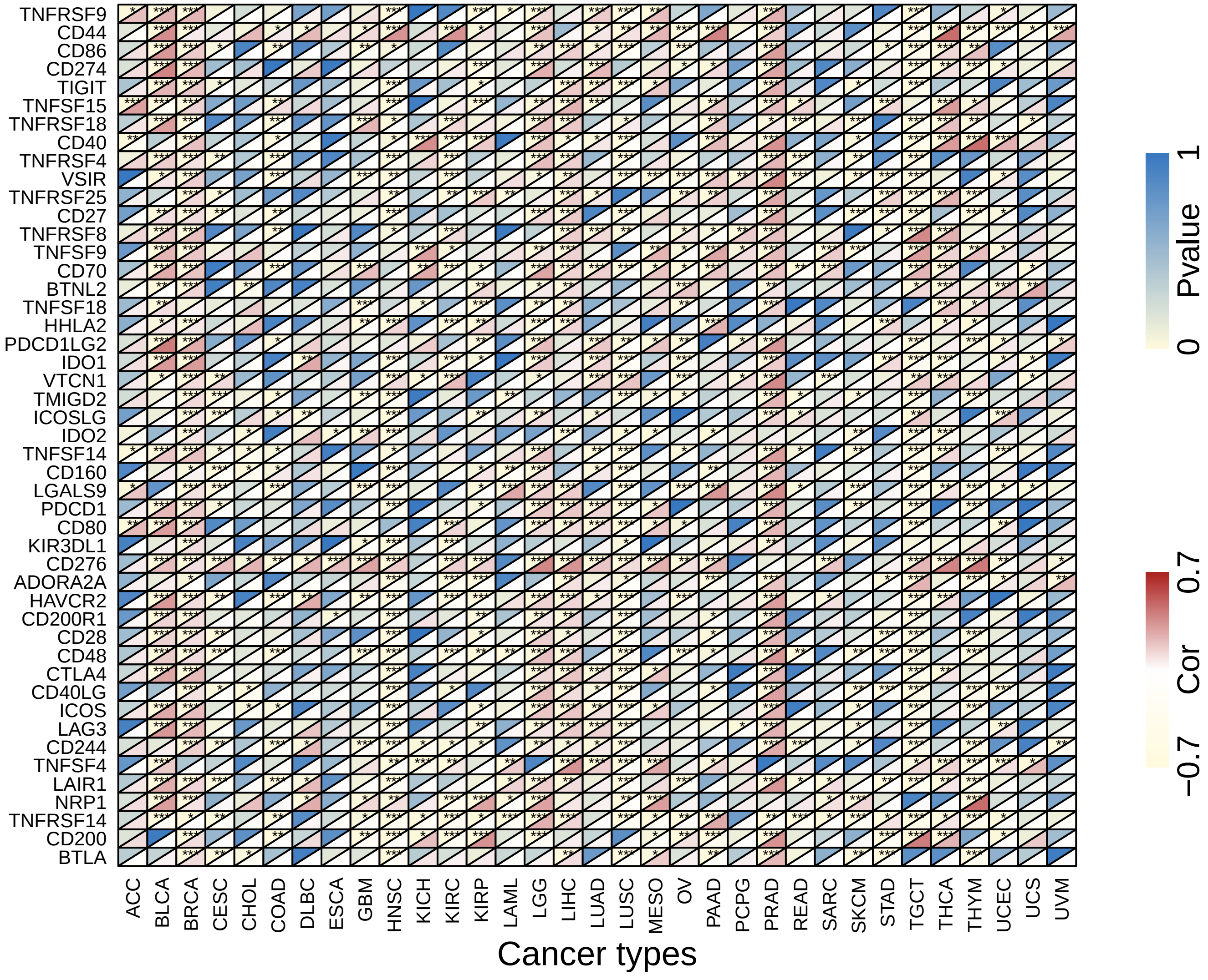

Supplement: Supplementary file 3 [file DataSheet_3.zip › Σ╗úτáü/IGF2BPs heatmap.zip!/IGF2BP2 heatmap/2heatmap.pdf]
