# Supplementary material for: A pan-cancer landscape of IGF2BPs and their association with prognosis, stemness and tumor immune microenvironment
Source: Front Oncol. 2023 Jan 4;12:1049183. doi: 10.3389/fonc.2022.1049183 (PMC9846525; doi:10.3389/fonc.2022.1049183)
Supplement: Supplementary file 3 [file DataSheet_3.zip › Σ╗úτáü/IGF2BPs heatmap.zip!/IGF2BP2 MSIradar/radar.pdf]

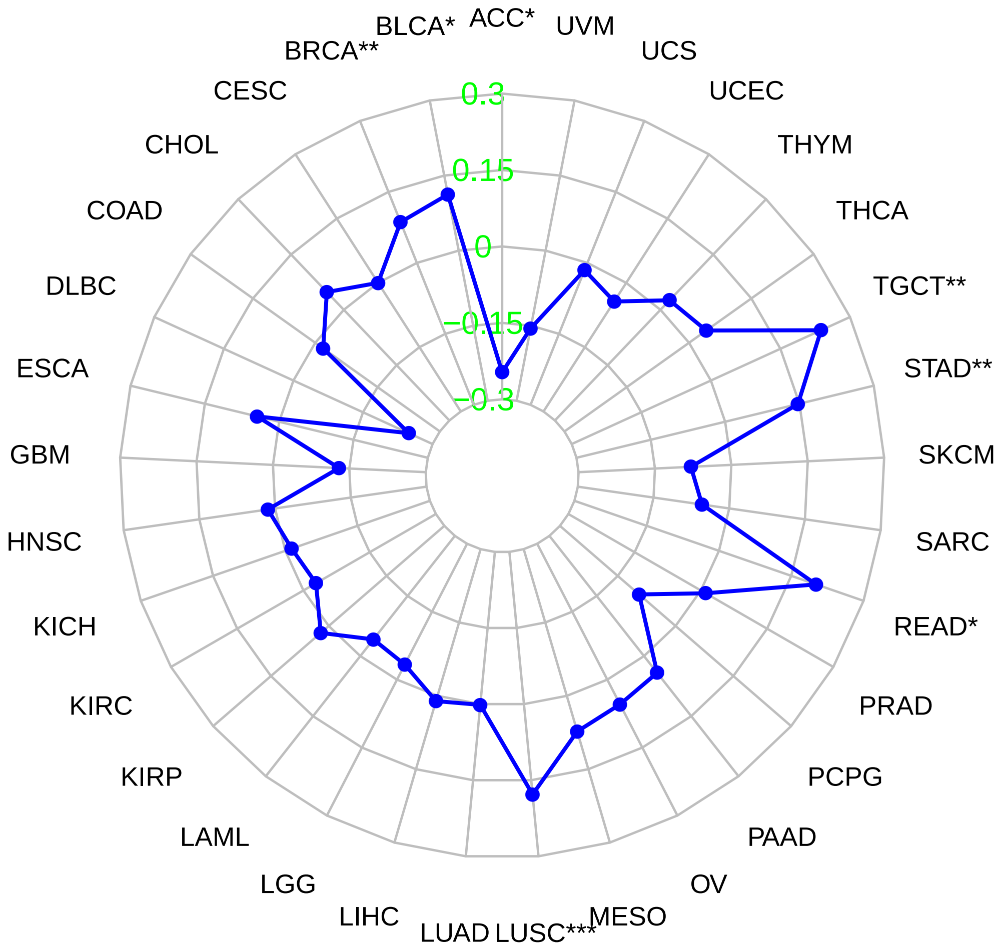
ACC*
UVM
UCS
UCEC
THYM
THCA
TGCT**
STAD**
SKCM
SARC
READ*
PRAD
PCPG
PAAD
OV
MESO
LUSC***
LUAD
LIHC
LGG
LAML
KIRP
KIRC
KICH
HNSC
GBM
ESCA
DLBC
COAD
CHOL
CESC
BRCA**
BLCA*
0.3
0.15
0
−0.15
−0.3
