# Supplementary material for: A pan-cancer landscape of IGF2BPs and their association with prognosis, stemness and tumor immune microenvironment
Source: Front Oncol. 2023 Jan 4;12:1049183. doi: 10.3389/fonc.2022.1049183 (PMC9846525; doi:10.3389/fonc.2022.1049183)
Supplement: Supplementary file 3 [file DataSheet_3.zip › Σ╗úτáü/IGF2BPs heatmap.zip!/IGF2BP2 TMBradar/radar.pdf]

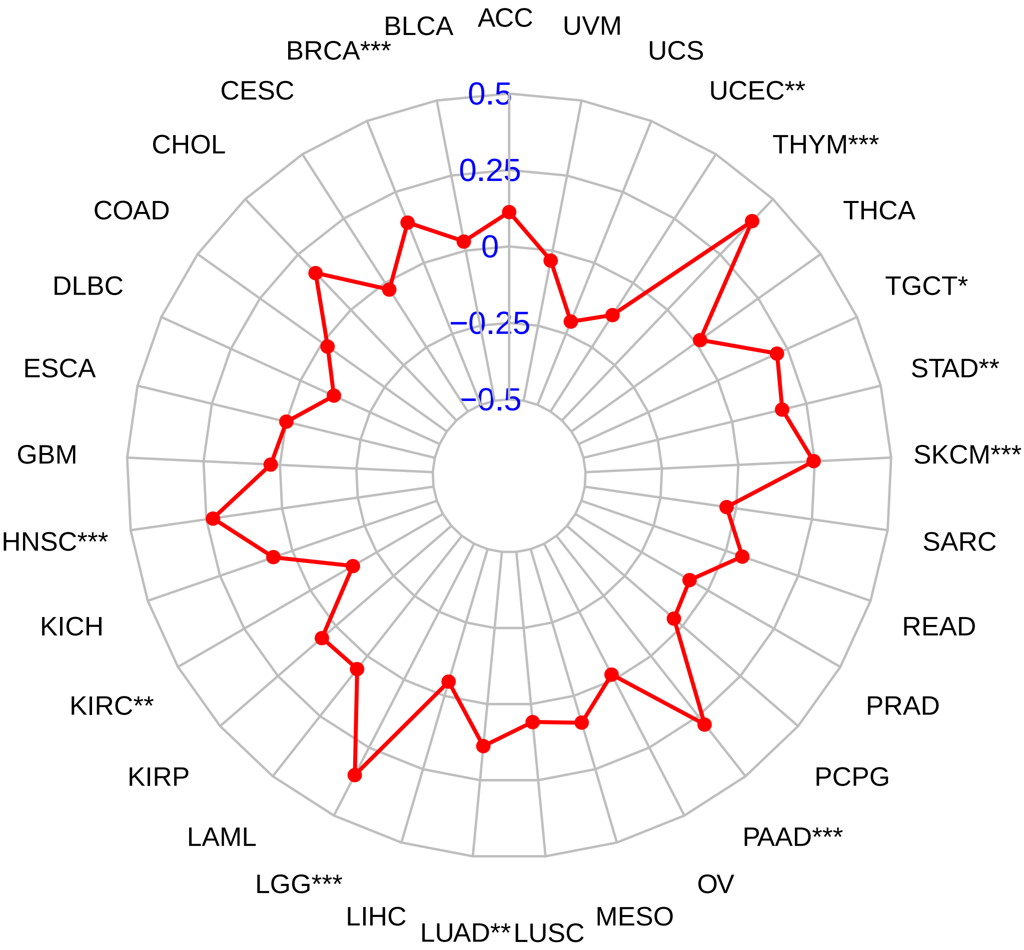
ACC
UVM
UCS
UCEC**
THYM***
THCA
TGCT*
STAD**
SKCM***
SARC
READ
PRAD
PCPG
PAAD***
OV
MESO
LUSC
LUAD**
LIHC
LGG***
LAML
KIRP
KIRC**
KICH
HNSC***
GBM
ESCA
DLBC
COAD
CHOL
CESC
BRCA***
BLCA
0.5
0.25
0
−0.25
−0.5
