# Supplementary material for: A pan-cancer landscape of IGF2BPs and their association with prognosis, stemness and tumor immune microenvironment
Source: Front Oncol. 2023 Jan 4;12:1049183. doi: 10.3389/fonc.2022.1049183 (PMC9846525; doi:10.3389/fonc.2022.1049183)
Supplement: Supplementary file 3 [file DataSheet_3.zip › Σ╗úτáü/IGF2BPs heatmap.zip!/IGF2BP3 MSIradar/radar.pdf]

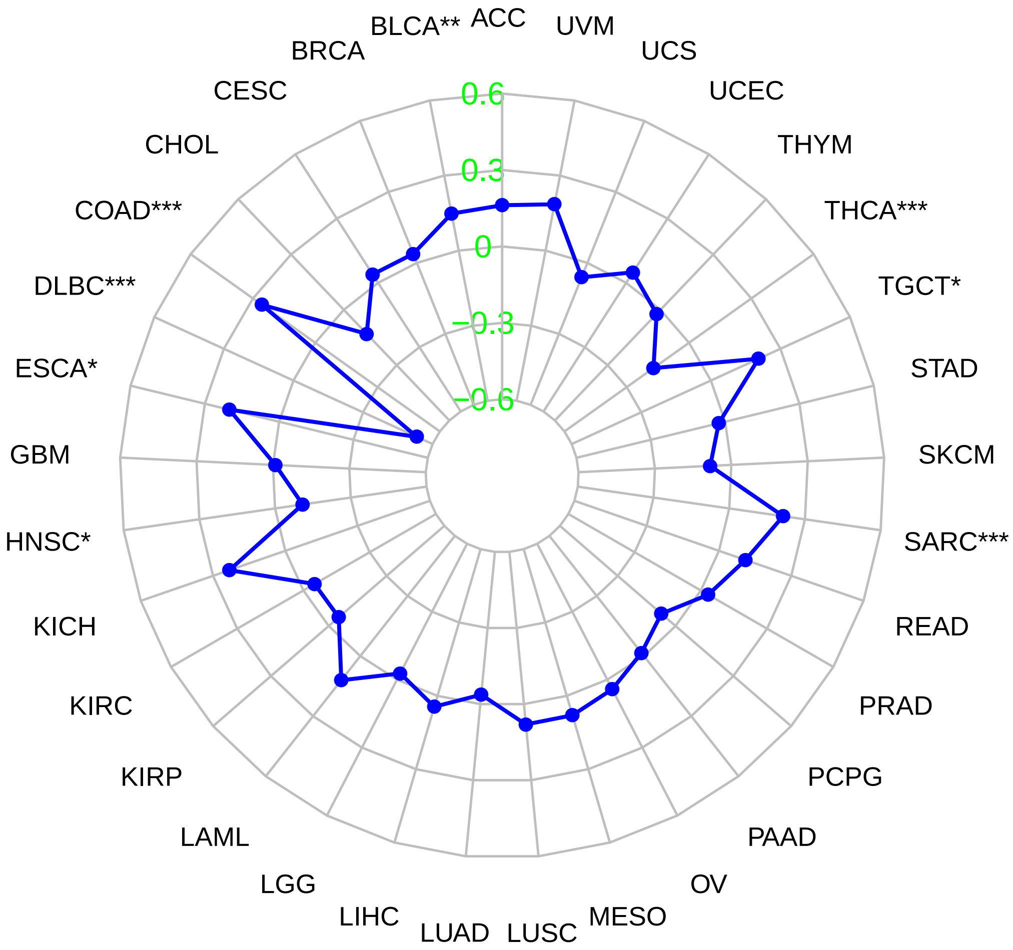
ACC
UVM
UCS
UCEC
THYM
THCA***
TGCT*
STAD
SKCM
SARC***
READ
PRAD
PCPG
PAAD
OV
MESO
LUSC
LUAD
LIHC
LGG
LAML
KIRP
KIRC
KICH
HNSC*
GBM
ESCA*
DLBC***
COAD***
CHOL
CESC
BRCA
BLCA**
0.6
0.3
0
−0.3
−0.6
