# Supplementary material for: A pan-cancer landscape of IGF2BPs and their association with prognosis, stemness and tumor immune microenvironment
Source: Front Oncol. 2023 Jan 4;12:1049183. doi: 10.3389/fonc.2022.1049183 (PMC9846525; doi:10.3389/fonc.2022.1049183)
Supplement: Supplementary file 3 [file DataSheet_3.zip › Σ╗úτáü/IGF2BPs heatmap.zip!/IGF2BP3 TMBradar/radar.pdf]

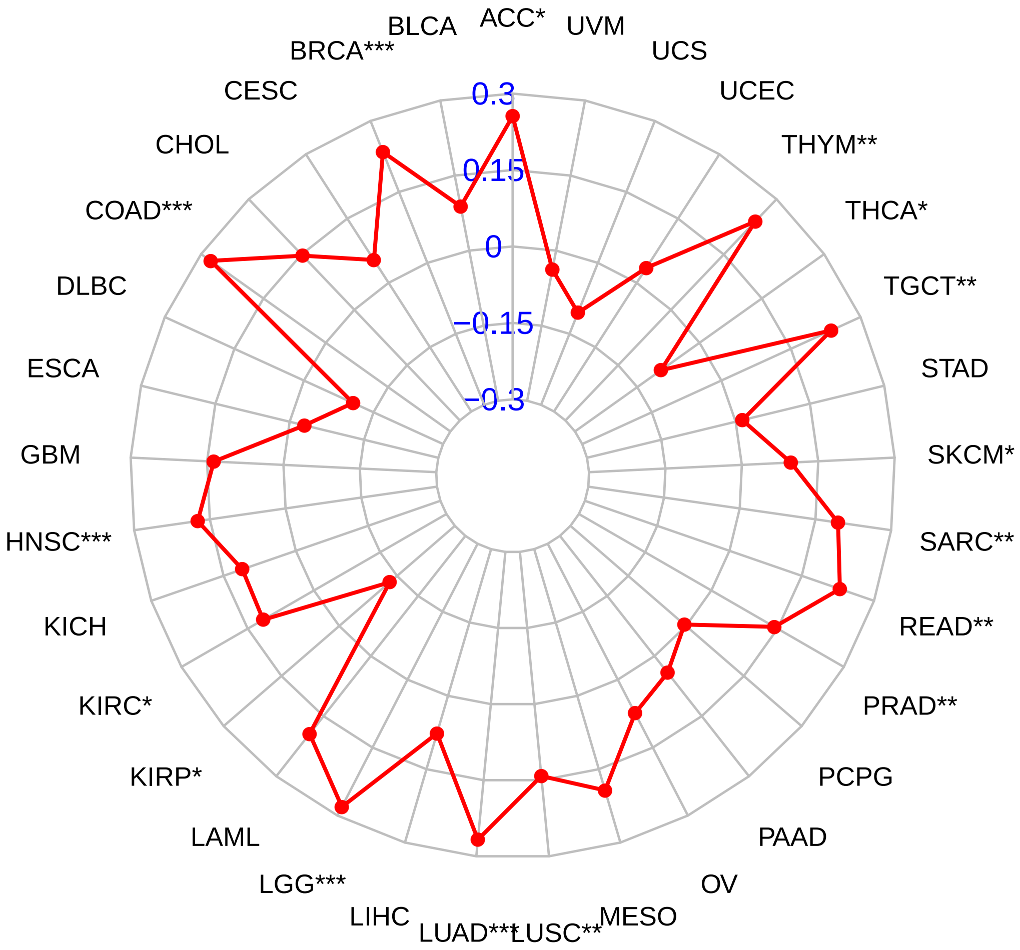

ACC*
UVM
UCS
UCEC
THYM**
THCA*
TGCT**
STAD
SKCM*
SARC**
READ**
PRAD**
PCPG
PAAD
OV
MESO
LUSC**
LUAD***
LIHC
LGG***
LAML
KIRP*
KIRC*
KICH
HNSC***
GBM
ESCA
DLBC
COAD***
CHOL
CESC
BRCA***
BLCA
0.3
0.15
0
−0.15
−0.3
