# Supplementary material for: A pan-cancer landscape of IGF2BPs and their association with prognosis, stemness and tumor immune microenvironment
Source: Front Oncol. 2023 Jan 4;12:1049183. doi: 10.3389/fonc.2022.1049183 (PMC9846525; doi:10.3389/fonc.2022.1049183)
Supplement: Supplementary file 3 [file DataSheet_3.zip › Σ╗úτáü/σ╜Æμíú.zip!/18.CellMiner/drugCor.pdf]

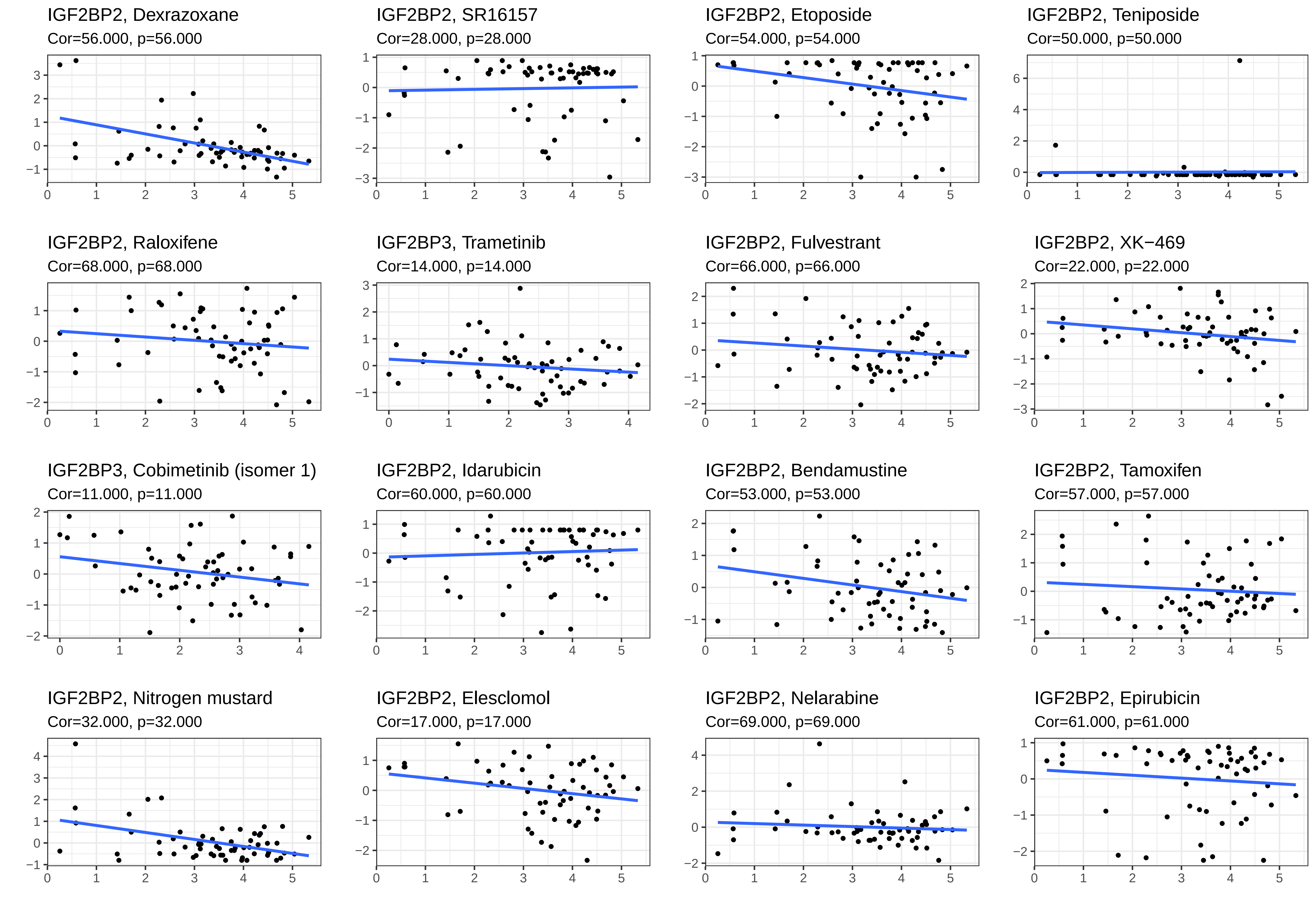

IGF2BP2, Dexrazoxane
Cor=56.000, p=56.000
IGF2BP2, SR16157
Cor=28.000, p=28.000
IGF2BP2, Etoposide
Cor=54.000, p=54.000
IGF2BP2, Teniposide
Cor=50.000, p=50.000
IGF2BP2, Raloxifene
Cor=68.000, p=68.000
IGF2BP3, Trametinib
Cor=14.000, p=14.000
IGF2BP2, Fulvestrant
Cor=66.000, p=66.000
IGF2BP2, XK−469
Cor=22.000, p=22.000
IGF2BP3, Cobimetinib (isomer 1)
Cor=11.000, p=11.000
IGF2BP2, Idarubicin
Cor=60.000, p=60.000
IGF2BP2, Bendamustine
Cor=53.000, p=53.000
IGF2BP2, Tamoxifen
Cor=57.000, p=57.000
IGF2BP2, Nitrogen mustard
Cor=32.000, p=32.000
IGF2BP2, Elesclomol
Cor=17.000, p=17.000
IGF2BP2, Nelarabine
Cor=69.000, p=69.000
IGF2BP2, Epirubicin
Cor=61.000, p=61.000
